# Exchange-induced spin polarization in a single magnetic molecule junction

**DOI:** 10.1038/s41467-022-31909-w

**Published:** 2022-08-03

**Authors:** Tian Pei, James O. Thomas, Simen Sopp, Ming-Yee Tsang, Nicola Dotti, Jonathan Baugh, Nicholas F. Chilton, Salvador Cardona-Serra, Alejandro Gaita-Ariño, Harry L. Anderson, Lapo Bogani

**Affiliations:** 1 Department of Materials, University of Oxford, 16 Parks Road, Oxford, OX1 3PH UK; 2 Department of Chemistry, University of Oxford, Chemistry Research Laboratory, Oxford, OX1 3TA UK; 3 Institute for Quantum Computing, University of Waterloo, 200 University Ave., N2L 3G1 Waterloo, ON Canada; 4 Department of Chemistry, School of Natural Sciences, University of Manchester, Oxford Road, Manchester, M13 9PL UK; 5 Instituto de Ciencia Molecular, Universidad de València, 2 C/Catedrático José Beltrán, Paterna, Valencia, Spain

**Keywords:** Electronic devices, Magnetic properties and materials

## Abstract

Many spintronic devices rely on the presence of spin-polarized currents at zero magnetic field. This is often obtained by spin exchange-bias, where an element with long-range magnetic order creates magnetized states and displaces the hysteresis loop. Here we demonstrate that exchange-split spin states are observable and usable in the smallest conceivable unit: a single magnetic molecule. We use a redox-active porphyrin as a transport channel, coordinating a dysprosium-based single-molecule-magnet inside a graphene nano-gap. Single-molecule transport in magnetic field reveals the existence of exchange-split channels with different spin-polarizations that depend strongly on the field orientation, and comparison with the diamagnetic isostructural compound and milikelvin torque magnetometry unravels the role of the single-molecule anisotropy and the molecular orientation. These results open a path to using spin-exchange in molecular electronics, and offer a method to quantify the internal spin structure of single molecules in multiple oxidation states.

## Abstract (web summary)

The spin exchange, which is central to spintronics, has been restricted to devices with long-range magnetic ordering to date. Here, Pei et al. design a single-molecule-magnet and utilize its internal spin exchange to control the current through a single-molecule junction with high spin polarization (>95%).

## Introduction

Molecular spintronics deals with electronic devices in which the active component is a single magnetic molecule so that electronic transport is controlled by the quantum spin states of just one molecular unit^1–6^. This approach has mainly focused on the long spin-flip times of single-molecule-magnets (SMMs) but questions about the interactions between flowing electrons and localized spin centers remain unanswered: Can intramolecular spin–spin interactions be used to operate a spintronic device? How can spin-exchange interactions be used to spin-polarize the electron flow?

**Fig. 1:**
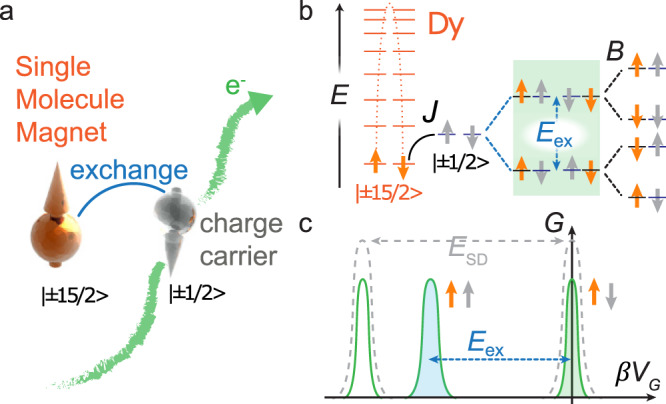
Exchange-induced polarization in molecular spintronics. **a** Intramolecular exchange scheme between the spin of a charge carrier in a localized conduction channel (gray) and a communicating single-molecule magnet (orange). **b** Implementation in **DyP**: the Dy(III) spin center (orange) is subject to an axial magnetic anisotropy and couples to an unpaired electron present on the porphyrin radical cation (gray). The intramolecular exchange coupling *J* splits the degeneracy of the conduction channel by the exchange energy, *E*_ex_, even in zero magnetic fields (green box), application of *B* field lifts the residual degeneracy. **c** The peaks in the differential conductance vs. gate potential are spaced by the source-drain energy (gray) due to the conduction channel entering and exiting the source-drain window. The exchange induces a splitting of one peak into two spin-polarized channels (blue and green) that are separated by intramolecular exchange energy, *E*_ex_, if the gate voltage, *V*_G_, is scaled by the gate lever arm, β. The two channels remain perfectly spin-polarized in *B* = 0 for a slow-relaxing SMM.

Although exchange interactions inside nanostructures have been investigated in lateral double-quantum-dots^7^ and carbon nanotubes^8^, the exploitation of intramolecular exchange remains largely uninvestigated in single-molecule electronics. Exchange-bias can shift the zero-field tunneling of SMMs^9^, and magnetic molecules have been used to pin inorganic ferromagnetic layers^10^ or to interface with surface states^11,12^. While exchange interactions lie at the foundation of exchange-bias effects^13–15^, single-molecule devices are, intrinsically, precluded from using long-range order to achieve exchange-bias. Previous results on phthalocyanine-rare-earth double-decker complexes and carbon nanotubes^16^ have shown the possibility of observing nuclear spin states^17,18^ and of reading spin-polarized states through intramolecular exchange^19^. However, these observations did not allow two differently polarized transport channels to be resolved, and thus no significant spin polarization of the conduction electrons was achieved. Here we demonstrate that internal spin–spin exchange can be used to achieve >95% spin-polarization, splitting differently polarized conduction channels in single-molecule devices. The concept of the device is the following: intramolecular spin exchange between an anisotropic metal ion and an isotropic conduction channel (Fig. 1a) splits the conduction levels into spin up and down for a given SMM orientation, even in the absence of a magnetic field (Fig. 1b)^17,19^. When only one transport channel is present, the intramolecular exchange will lead to an additional peak in the zero-field differential conductance, when plotted as a function of gate potential, *V*_G_ (Fig. 1c), due to the spontaneous separation of the transport channel into spin-up and down spin channels of the device.

In weakly-coupled single-molecule junctions, electron transport happens via sequential charging events, and not via a continuous electron flow. Therefore an experimental realization of a single-molecule device exploiting exchange interactions is challenging: electron passage needs to affect only one part of the molecule, while the magnetic state of the other part remains unaffected by the sequential addition/removal of electrons. To this aim, we need to design an SMM with two moieties: one through which sequential transport occurs, and a magnetic one that splits the spin-up and spin-down channels of the former via an exchange interaction, without undergoing oxidation or reduction itself. Another requirement for designing unimolecular devices with highly polarized spin currents is that thermal- and lifetime-broadenings must be lower than the intramolecular exchange. This requirement enforces that our devices must operate in the weakly-coupled transport regime. The often-used bonding to gold leads (usually via thiols) produces molecule-electrode couplings of a few to tens of meV^1,5,20,21^, higher than the typical intramolecular exchange. Coupling to graphene electrodes via *π*-stacking interactions^22,23^ is usually well below 1 meV, thus providing access to sufficiently weakly-coupled devices such that exchange-induced effects can be resolved^19^.

## Results

### Molecular design and device fabrication

**Fig. 2:**
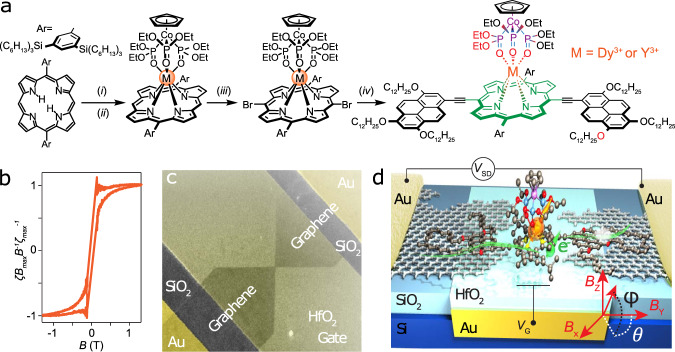
The molecules and the nanodevices. **a** Scheme of the synthesis of the rare-earth porphyrin molecular complex, **MP**. **M** can be Y^3+^ (no exchange coupling) or Dy^3+^ (introducing the spin exchange). The redox-active porphyrin and the metal center are highlighted in green and orange, respectively. Reagents and conditions: (i) MCl_3_·6H_2_O, imidazole, benzamidazole, Ph_2_O, 200 °C, 16 h; (ii) Na[CpCo{P(O)(OEt)_2_}_3_], (iii) *N*-bromosuccinimide, CH_2_Cl_2_; (iv) 1,3,6-*tris*(dodecyloxy)-8-ethynylpyrene, Pd(PPh_3_)_4_, CuI, DIPA, 65 °C, 16 h. **b** Hysteresis cycle detected by cantilever-torque magnetometry at 150 mK with a field-sweep rate of 0.125 T/min, showing single-molecule-magnet behavior for **DyP** derivative. The torque signal ζ is divided by the applied magnetic field *B* and normalized by the high-field signal ζ_max_/*B*_max_. **c** False-color SEM image of the devices, showing Au pads (yellow), the Au/HfO_2_ back-gate (gray), and the graphene electrodes (darker shade). **d** Scheme of the experiment on **DyP** and the reference frame of the magnetic field *B* (red). The Dy(III) spin center (orange) sits on the redox-active porphyrin, which spans the nanogap and exchanges electrons (green) via pyrene anchor groups.

With these principles in mind, we designed the molecule **MP** (Fig. 2a), which consists of a trivalent rare-earth ion (**M**), coordinated by a redox-active porphyrin (**P**) functionalized with two electron-rich pyrene anchor groups, as an electronically-separate spin center that can couple to the graphene via *π*-stacking. Porphyrin ligands differ from the more commonly used phthalocyanines in that they allow functionalization at the *meso* positions, which confers stronger electronic coupling with the *π*-system than *β*-functionalization and is more effective for controlling aggregation. This molecular design has been shown to be effective for bridging graphene nanogaps^22^. Sequential electron transport occurs via the *π*-system of the porphyrin moiety, which can be easily reduced and oxidized, while the redox potentials of the rare-earth are inaccessible within the experimental gate-voltage window^24,25^. The harsh reaction conditions required to insert the lanthanide cation into the porphyrin are incompatible with the ethynylpyrene anchor groups, so we coordinate the porphyrin before bromination and use Sonogashira coupling to attach the anchor groups (Fig. 2a, synthetic procedure in Supplementary Methods). Isostructural compounds are obtained by using different MCl_3_ salts^26,27^, thus allowing insertion of diamagnetic Y(III) (to give **YP**) and magnetic Dy(III) (to give **DyP**), and comparison of the transport properties in the absence and presence of exchange coupling. The metal ion is coordinated via four nitrogen atoms of the porphyrin, and three oxygens of the Kläui cap^28^, producing a low-symmetry hepta-coordinate metal center. For **DyP**, the coordination geometry produces a strong magnetic anisotropy and a well-defined direction of the magnetic anisotropy of the spin center^26^, with the principal magnetization axis along the Dy-Co direction (vide infra). The high anisotropy of the Dy(III) ion leads to an exponential decrease of the spin-flip time on lowering the temperature, *T*, and single-molecule-magnet behavior: when the magnetic field *B* is swept, the magnetization of the molecule relaxes slower than the sweeping rate, and single molecules display magnetic hysteresis without requiring any three-dimensional ordering^2,5,9,26,27^. This hysteresis is indeed observed at temperatures comparable to the transport experiments using a torque magnetometer (Fig. 2b) and the full angular dependence of the anisotropy, as a function of the orientation of the magnetic field can be extracted (detailed in Methods section).

Fabrication of the devices follows a published procedure^22^ that is described in the Methods section. In brief, graphene ribbons are patterned lithographically into a bowtie shape and feedback-controlled electro-burning forms graphene nanogaps that are 1–2 nm, as estimated from the tunneling currents (Fig. 2c)^29^. Single-molecule transistors are then produced by drop-casting a solution of **MP** in toluene (2 µM) onto the nanogaps. A local gate electrode, separated by 10 nm of HfO_2_, runs under the tunnel junction allowing the molecular levels to shift with respect to the chemical potential of the leads by a gate voltage *V*_G_ (Fig. 2c, d) and modulating the current *I*_SD_ produced by the source-drain voltage *V*_SD_.

### Charge transport measurements

**Fig. 3:**
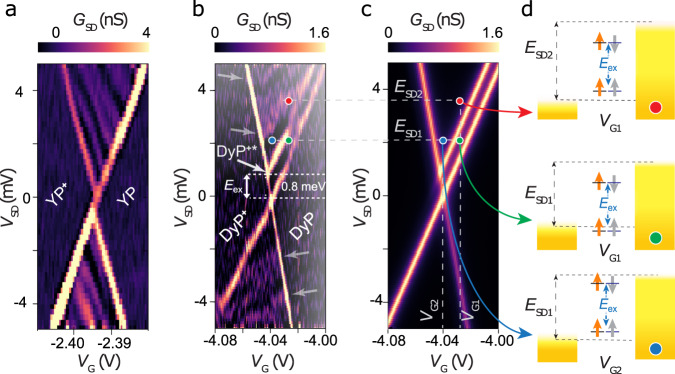
Single-molecule transport measurements. **a** Map of the differential conductance ${G}_{{SD}}=\partial {I}_{{SD}}/\partial {V}_{{SD}}$, vs. *V*_G_ and *V*_SD_ for **YP**, at 20 mK, at *B* = 0. Coulomb diamonds are labeled with molecular states. **b** *G*_SD_ map of the **DyP**^***+***^**/DyP** transition at *B* = 0 and 20 mK. Gray arrows indicate interference from graphene leads. The excited spin state, **DyP**^+^* is visible (white arrow) and marks indicate representative transport regions. **c** Simulation of the differential conductance map for the **DyP**^**+**^**/DyP** transition. Marks indicate representative transport regions. **d** Transport channels are available in the different regions, with the molecular energy levels depicted between the electrode energies (yellow). Arrows indicate the ferro- and antiferromagnetic arrangements of the Dy (orange) and flowing electron spin (gray).

Figure 3a, b and Supplementary Fig. 26 show the stability diagrams of the differential conductance ${G}_{{SD}}=\partial {I}_{{SD}}/\partial {V}_{{SD}}$ vs. *V*_SD_ and *V*_G_ for several **MP** devices. The observation of Coulomb blockade confirms that graphene-**MP** molecular devices operate in the desired weakly-coupled transport regime, with $\Delta E\gg {k}_{{{{{{\rm{B}}}}}}}T,\Gamma$, where $\Delta$*E* is the energy spacing between molecular levels, *k*_B_ is the Boltzmann constant and $\Gamma ={\Gamma }_{{{{{{\rm{S}}}}}}}+{\Gamma }_{{{{{{\rm{D}}}}}}}$ is the electronic coupling to the source and drain electrodes^24,30^. For weakly-coupled molecular junctions, single-electron tunneling is the mechanism of charge transport, with regions of blocked current produced by Coulomb repulsion where the charge on the molecule is fixed. Between these regions, current flows through the weakly-coupled molecular junction as a result of sequential electron transfer to (i.e., a reduction process) and from (i.e., an oxidation process) the molecule. Within sequential tunneling regions, several lines of higher conductance are visible, running parallel to the diamond edges or slanted: parallel lines are molecular states, while slanted lines correspond to interference inside graphene leads, unrelated to molecular behavior^31^. Our measurement conditions impose that $\Delta E \, > \, \Gamma ,{k}_{{{{{{\rm{B}}}}}}}T$, so that *Γ* can be determined^32^ as the full-width half-maximum of *G*_SD_ vs. *V*_G_ at *V*_SD_ = 0 mV (Supplementary Fig. 27). For the **YP** and **DyP** devices presented in Fig. 3 we obtain *Γ* = 0.30 meV (**YP**) and 0.34 meV (**DyP**). As ${k}_{{{{{{\rm{B}}}}}}}T$ ~2 µeV at 20 mK, this indicates lifetime broadening due to $\Gamma$, rather than thermal broadening, is the main contribution to the signal broadening. The electrostatic coupling between the gate potential and the molecular states is given by the gate lever arm, *β*, (0.23 for **YP** and 0.085 for **DyP**, Supplementary Fig. 27) and this allows us to convert between *V*_G_ and the intrinsic energy scales of the molecule. The variation in *β* is attributed to imperfections in the gate dielectric or different screening of the gate-field by the source and drain electrodes and is in-line with previous reports on graphene single-molecule devices^22,33^.

**Fig. 4:**
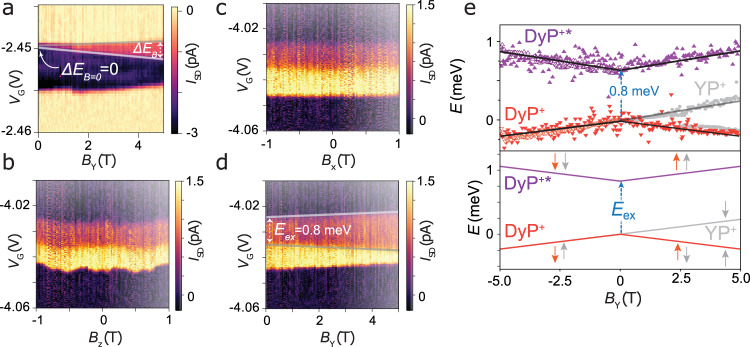
Exchange splitting. **a** Splitting of the lowest energy peak for the **YP** device, on increasing *B*_Y_, acquired at *V*_SD_ = –1.5 mV. A spin doublet is visible (gray lines), without exchange. **b** Dependence of the ground state and excited state transition potentials for **DyP**^**+**^**/DyP** on increasing *B*_z_, acquired at *V*_SD_ = 1.5 mV. **c** Dependence of ground state and excited state transition potentials for **DyP**^**+**^**/DyP** on increasing *B*_x_, acquired at *V*_SD_ = 1.5 mV. **d** Dependence of ground state and excited state transition potentials for **DyP**^**+**^**/DyP** on increasing *B*_y_, acquired at *V*_SD_ = 1.5 mV. **e** Upper panel shows a separation of the magnetic levels vs. *B*_Y_, for two **DyP** devices (triangles, full and open) and for the **YP** device (gray circles). Lines are fits to the data. Lower panel, expected evolution of the magnetic field levels for **DyP**^**+**^ (color) and **YP**^**+**^ (gray). Dy(III) spins are orange and porphyrin ones gray. All data were acquired at 20 mK.

By choosing *V*_G_, the molecule can be fixed in the **MP**, **MP**^**+**^, or **MP**^**–**^ oxidation state, (see Supplementary Discussion and Supplementary Fig. 28 for details of the assignment) due to Coulomb blockade^24,34^. The transport regions that are studied in detail (Fig. 3a, b) are the **MP**^**+**^/**MP** transitions. The creation of **MP**^**+**^ by removing an electron from the redox-active porphyrin leads to a state that contains, in addition to the M(III) center, a spin-1/2 radical delocalized inside the porphyrin π-system. We first investigate the molecular system without exchange interactions, **YP**. A magnetic field *B* splits the **YP**^**+**^**/YP** transition into two diverging states, corresponding to the spin-up and spin-down states of the electrons flowing through the porphyrin channel (Fig. 4a). The states are separated by $\Delta E=g{\mu }_{{{{{{\rm{B}}}}}}}B=\beta \Delta {V}_{{{{{{\rm{G}}}}}}}$, where ${\mu }_{{{{{{\rm{B}}}}}}}$ is the Bohr magneton, and *g* is the Landé factor of the *s* = 1/2 porphyrin radical in the **YP**^**+**^ state. As expected for a Kramers doublet, no splitting is present at *B* = 0 and the fitted *g* = 1.7 ± 0.2 matches that expected for an electron spin in a porphyrin ring (*g* ~2)^34^ (Fig. 4e, gray points).

In **DyP**, where the intramolecular exchange is introduced, a new molecular state appears at 0.8 ± 0.1 meV above **DyP**^**+**^**/DyP** (Fig. 3b). The excited state meets the Coulomb diamond edge on the **DyP**^**+**^ side, indicating that this is an excitation of the cationic state **DyP**^**+**^, rather than of **DyP**, and we shall thus call it **DyP**^+^*. The evolution of the **DyP**^**+**^**/DyP** and **DyP**^+^*/DyP transitions, as a function of the magnitude and direction of *B*, are very rich (Fig. 4b–d). The behavior perpendicular to the plane of the graphene, *B*_Z_, is strongly nonlinear, with a pronounced and symmetrical displacement about *B*_Z_ = 0. In contrast, along *B*_X_ the transitions remain largely independent of field strength in the range –1 T < *B*_X_ < 1 T. We attribute this to the rough alignment of the molecular easy axis normal to the graphene plane (vide infra for a more detailed discussion of the anisotropy). The two **DyP**^+^* and **DyP**^**+**^ levels clearly diverge vs. increasing |*B*_Y_|, indicating that they must have different spin states (Fig. 4d).

Figure 4e shows the *B*_Y_-dependence of the energies of the **DyP**^**+**^ and **DyP**^+^* transitions, for both positive and negative *B*_Y_, for different **DyP** devices, and compared to that of **YP**. The electronic transport and the spin splitting are reproducibly observed in different devices and among different chips and is consistent whatever the sign of *B*_Y_. The slope of the divergence is consistent with the **YP** data, and electronic Landé *g* factors of 1.7 ± 0.4 and 1.9 ± 0.2 are measured for two **DyP** devices, compatible with the sensing of the electron spin localized on the porphyrin moiety. The 0.8 meV level splitting in *B*_Y_ = 0, which is visible only for **DyP** devices, cannot arise from electric field effects or transverse anisotropy terms: Kramers parity theorem strictly forbids any zero-field-splitting for both the unpaired porphyrin spin and the Dy(III) ion. On the contrary, the splitting is a fingerprint of the intramolecular exchange interaction, as outlined in the following paragraph.

For **DyP**^**+**^ the porphyrin spin *s* = ½ is roughly-isotropic, while Dy(III) is a highly anisotropic ^6^H_15/2_ center, with a spin-orbit-coupled angular momentum *J* = 15/2. The spin Hamiltonian of the system is thus:

1 $$\hat{H}=\hat{s}{{{{{\bf{J}}}}}}\hat{J}+\hat{s}{{{{{\bf{D}}}}}}\hat{J}+{\mu }_{B}\hat{B}{{{{{\bf{G}}}}}}\hat{J}+{\mu }_{B}\hat{B}{{{{{\bf{g}}}}}}\hat{s}+{\sum }_{k=2,4,6}\mathop{\sum }\limits_{q=-k}^{k}{B}_{{kq}}{\hat{O}}_{k}^{q}$$

where **J** is the exchange interaction tensor, **D** the dipolar spin–spin coupling tensor, **G** and **g** are the Landé tensors for Dy and the porphyrin, respectively, and the sum contains Stevens operator equivalents, i.e., a sum with weights ${B}_{{ij}}$ over the tesseral harmonics operators ${\hat{O}}_{i}^{j}$. The Stevens operators, because of the high value of ${B}_{20}$ in Dy systems, split Dy(III) spin levels into a series of different Kramers doublets even at zero-field (Fig. 1b). At *T* < 5 K we can consider only the lowest-lying Kramer’s doublet and Dy(III) behaves as an effective spin ${S}_{{{{{{\rm{eff}}}}}}}=\frac{1}{2}$ with a large effective Landé factor ${G}_{{{{{{\rm{eff}}}}}}} \sim 18$^26^. We can therefore identify the different transitions observed in the experimental data to different spin states of **DyP**^**+**^ using the assignment: $|{m}_{s},{m}_{{S}_{{{{{{\rm{eff}}}}}}}}\rangle$. The ground state **DyP**^**+**^**/DyP** transition (occurring at zero bias voltage) is via the antiferromagnetic $|\!-\!1/2,1/2\rangle$ and $|1/2,-1/2\rangle$ state of **DyP**^**+**^ **(**hereafter indicated with↑↓). The excited state **DyP**^+*^/DyP transition at 0.8 ± 0.1 meV above the ground state transition is via the ferromagnetic (↑↑) $|1/2,1/2\rangle$, $|\!-\!1/2,-1/2\rangle$ state of the cation, with the energy splitting corresponding to the exchange energy, *E*_ex_. Excellent agreement is obtained between the resulting energy vs. *B*_Y_ evolution and the experimental data, for an antiferromagnetic exchange interaction, between ${S}_{{{{{{\rm{eff}}}}}}}$ and *s*, *J* = –2 *E*_ex_ = –1.6 ± 0.2 meV (Fig. 4e). The **YP** system is much simpler by design. The electron configuration of Y(III) is closed-shell [Kr], so there are no unpaired spins or unquenched orbital angular momentum (*S* = 0, *L* = 0), and hence it functions as a control molecule because no intramolecular exchange interactions are present. The relevant spin states are solely due to the unpaired spin on the porphyrin of **YP**^**+**^, $|{m}_{{{{{{\rm{s}}}}}}}\rangle =|\pm 1/2\rangle$, and the only spin Hamiltonian term that remains accounts for Zeeman splitting of these (${\hat{H}=\mu }_{{{{{{\rm{B}}}}}}}\hat{B}{{{{{\boldsymbol{g}}}}}}\hat{s}$). This expected behavior is observed in the experimental data (Fig. 4a, e).

**Fig. 5:**
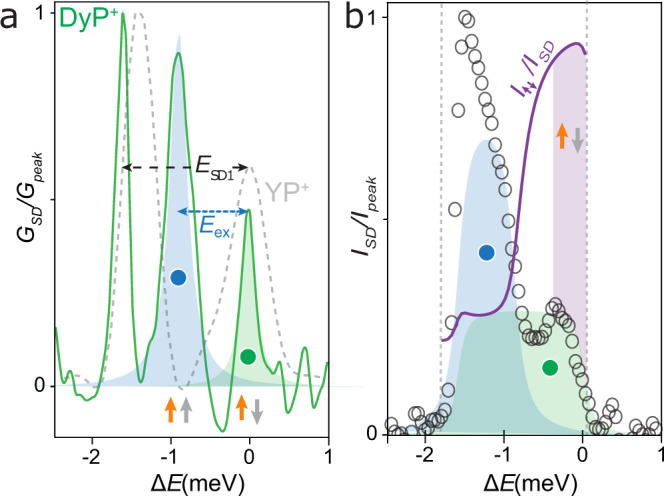
Molecular polarization effect. **a** Zero-field conductance of **YP**^**+**^ (gray) and **DyP**^**+**^ (green) devices at *E*_SD_ = 1.5 mV vs. the energy displacement [Δ*E* = β_MP_ (*V*_G_ – *V*_c_), where *V*_c_ is the gate voltage of the Coulomb peak]. The splitting of the conductance peak induced by the exchange is highlighted in blue. Curves are scaled for their maximum peak value, for clarity. Shaded areas are Lorentzian fits, and marks indicate the conductance mechanism as in Fig. 3, panel d. **b** Contributions to the current of **DyP**^**+**^ (gray circles) by the ferromagnetic (blue) and antiferromagnetic (green) molecular transport channels. Curves are scaled for their maximum peak value to compare to the fraction of spin-polarized electrons (violet). The violet region indicates a polarization higher than 90%, colored marks indicate the conductance mechanisms, as in Fig. 3.

A master-equation framework (outlined in Supplementary Discussion)^35^ allows the full stability diagram to be calculated, again in excellent agreement with the data of Fig. 3b (see Fig. 3c). Different conductance regions correspond to different states involved in the current (Fig. 3d). For a fixed orientation of Dy(III), two energy levels of **DyP**^**+**^, with a different spin on the porphyrin ring can both contribute to transport if the bias window is sufficiently large (top, red marker) or if the gate voltage is sufficiently negative (bottom, blue marker). Conversely, there is a region where transport occurs via one state of **DyP**^**+**^, corresponding to the antiferromagnetic spin configuration ${{{{{{\bf{DyP}}}}}}}_{\uparrow \downarrow }^{+}$ (middle, green marker). In this region only the electrons with the same spin as Dy(III) can tunnel from the HOMO of **DyP** to generate ${{{{{{\bf{DyP}}}}}}}_{\uparrow \downarrow }^{+}$, and only electrons of that same spin can tunnel into the SOMO of ${{{{{{\bf{DyP}}}}}}}_{\uparrow \downarrow }^{+}$, owing to Pauli exclusion. In this way the device allows the passage solely of conduction electrons with the same spin as Dy(III). The magnetometry data (Fig. 2b) confirms that at low or zero magnetic field, ${S}_{{{{{{\rm{eff}}}}}}}$ can be fixed, and as a consequence the molecule can act as a spin-polarizer. A small magnetic field initializes *S* to either orientation depending on the field-direction, and the intramolecular exchange splitting gives rise to a spin-polarized current of either ${m}_{s}=\frac{1}{2}$ or ${m}_{s}=-\frac{1}{2}$. The splitting of the conductance peak is clearly observable in its gate dependence for **YP**^**+**^ and **DyP**^**+**^ (Fig. 5a): the exchange splitting leads to the creation of an additional conductance peak in perfect agreement with the predictions of Fig. 1c. We can fit the conductance peaks to extract the separate contributions (*I*_↑↓_ and *I*_↑↑_) that occur via the different states of **DyP**^**+**^ to *I*_SD_ (Fig. 5b) (see Methods for the fitting procedure). As the intramolecular exchange interaction is large compared with the lifetime and thermal broadening the resulting current polarization ratio ${P}=(I_{\uparrow \downarrow }-{I}_{\uparrow \uparrow })/({I}_{\uparrow \downarrow }+{I}_{\uparrow \uparrow })$ is very high, and reaches 0.94 ± 0.35 for a *V*_G_ region of 5.4 mV even at *B* = 0. Due to the additional splitting of the peaks as a result of the Zeeman effect this region increases further in size to 8 mV with *B*_Y_ = 5 T (Supplementary Fig. 30) with a maximum of *P* = 0.97 ± 0.28.

### Magnetic anisotropy

**Fig. 6:**
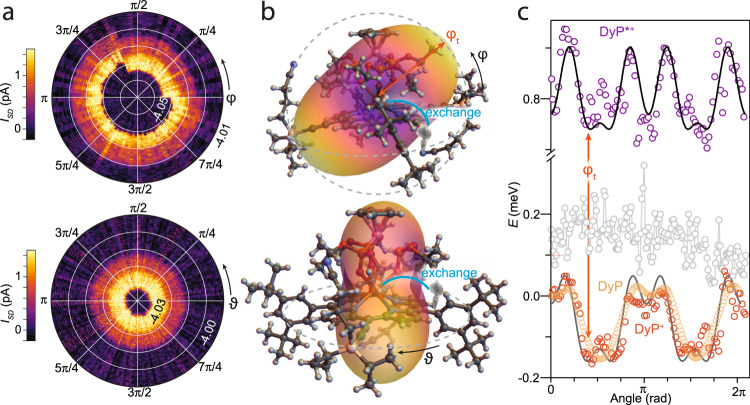
Angular relation to single-ion anisotropy. **a** Measured angular evolution of the exchange effect, showing the current (color scale) vs. *V*_G_ (radial) and the angle (top: out-of-plane φ; bottom: in-plane *θ*), under constant magnetic field-magnitude |*B* | = 1 T, at *V*_SD_ = 1.5 mV. A charge jump is observed for 0 < *φ* < 2π/3). **b** Iso-energy representation of the single-ion anisotropy of the Dy(III) ion, superimposed onto the molecular structure, as viewed from sideways in the single-molecule device architecture (top) and along the electron transport channel (bottom). **c** Angular evolution of the energy, relative to the ground state at zero angle, of the **DyP**^+^* (violet) and **DyP**^**+**^ (orange) states vs. *φ*, with the anisotropy energy extracted from torque measurements on the **DyP** at the same temperature superimposed (yellow). The lack of evolution vs. *θ* is also shown (gray). Black lines are fits to the data (see text). All data were acquired at 20 mK.

As exchange interactions are dependent on the mutual orientation of the spins, and can be extremely sensitive to the large anisotropy of rare-earth, they also offer a way to probe the orientation of the molecular easy axis via single-molecule experiments. The single-ion anisotropy will produce fingerprints in the magnetoconductance signal and modulate it as a function of the orientation of the magnetic field. We carried out a complete characterization of the **DyP**^**+**^ and **DyP**^+^* levels vs. the angles of *B*, in the plane of the graphene, *θ*, and out-of-plane, *φ* (Fig. 6a, b). No appreciable angular dependence is observed for *θ*, on the contrary, a non-monotonous angular dependence is observed for *φ*, with **DyP**^+^* displaying several local energy minima and maxima, with a periodic pattern (Fig. 6b). These observations, in combination with the variation with field strength (Fig. 3), lead to the conclusion that the axial anisotropy of the Dy(III) is roughly lying along the laboratory frame *z-*axis, perpendicular to the graphene plane. Calculation of the magnetic anisotropy of the molecular system with ab initio methods (see Methods) reveals that the magnetic anisotropy of the Dy(III) center is tilted to the plane of the porphyrin at an angle of ca. 70° (Fig. 6b and Supplementary Fig. 31). A much less structured angular evolution is expected when rotating *B* in the porphyrin plane along *θ*, owing to the axial anisotropy of the lowest Kramers doublet (Supplementary Table 1).

The angular dependence can be described, without loss of generality, as the sum: $E=\mathop{\sum }\nolimits_{n}^{N}{C}_{n}{{\cos (2n\varphi )}}$, where the *C*_*n*_ indicate the weights of the cosine terms (values in Supplementary Table 2), and the resulting fits in Fig. 6c show energy minima that indicate a tilted magnetic easy cone anisotropy of Dy(III) at a tilt angle of ${\varphi }_{{{{{{\rm{t}}}}}}}=68^\circ$. The correspondence of the tilt angle of the Dy(III) anisotropy measured within the lab frame and the ab initio calculated values within the porphyrin framework indicates the porphyrin plane is sitting roughly on the surface, which indeed is what is expected from the molecular design. Both the **DyP**^**+**^ and **DyP**^+^* levels display the same overall symmetry, as expected because they both arise from the exchange of Dy(III) with an unpaired electron on the porphyrin ring, but **DyP**^+^* displays much more pronounced energy oscillations at *φ* = 0, *π*, in agreement with an antiferromagnetic interaction.

We can now directly compare the single-molecule transport with angular-resolved CTM measurements on the neutral DyP system, performed in the same conditions at mK temperatures. The anisotropy energy that is extracted from torque measurements via the relation ${\zeta }_{z}=\delta E{\,}(\phi )/\delta {\,}\phi$ shows the presence of the same anisotropy signal in the *φ* dependence (Fig. 6c), with the difference that the neutral system lacks the unpaired spin in the porphyrin, as revealed by the signal around *φ* = *π*/2. By comparing the behavior of the single-molecule device and crystals, we can thus demonstrate that the single-ion anisotropy influences the magneto-resistive signal of transport electrons via the exchange interaction, and will modulate the transport characteristics of molecular spintronic devices.

## Discussion

These results demonstrate that the spin exchange internal to a molecule can be used to control the electron flow through it, and obtain high spin polarization in the tunneling current. The angular dependence provides a direct insight into the role of the molecular anisotropy on the modulation of the transport. Before any optimization, the resulting spin polarization, 94 ± 35%, already outclasses many bulks and single-molecule spin polarizers^36,37^. We use gate and field-dependent measurements at low temperatures to show how the interplay between the molecule-electrode coupling strength and the intramolecular exchange energy is crucial to obtain this high degree of polarization. The relevant internal energy scale, *J*, can reach up to hundreds of K in molecular magnets, so that polarization is conceivable at temperatures well above those used in this demonstration, especially when using graphene electrodes^29^. Our observations have several implications regarding the utility of such a highly polarized tunneling current. The possibility of hybrid devices can be explored, integrating spin detectors directly onto the graphene^38^ but far from the junction, to investigate the dependence of the µm long spin coherence length of graphene on the tunnel barriers and the presence of other magnetic molecules. Alternatively, modular porphyrin chemistry can be used to integrate a second porphyrin directly into the molecular structure^26^, such that the spin-polarized current injected into the first porphyrin can be read out by the second, creating a molecular spin valve. Integration of all the elements of a spintronic device in a single molecule, thus avoiding magnetic electrodes, is a desirable goal. The fundamental demonstration from this study that a lanthanide porphyrin acts as a highly effective spin-polarizer, is a key first step to create such a device. Furthermore, these results open completely new perspectives for the investigation of magnetic molecules, offering a new methodology to unravel the internal interaction patterns, even for different oxidation states and in non-crystalline environments.

## Methods

### Device fabrication

An array of local gate electrodes (Ti/Au, 40 nm thick) was patterned onto a doped Si/SiO_2_ substrate via photolithography. Second, a 10 nm layer of HfO_2_ was deposited by atomic layer deposition (ALD) to isolate the gate electrode. Then pairs of Ti/Au source and drain electrodes (60 nm thick) were added via a second photolithography step on top of the ALD layer. CVD-grown graphene was transferred onto the substrate using wet-transfer techniques and was etched into bowtie shapes using electron-beam-lithography and O_2_-plasma etching. A gap was opened in the graphene constriction using feedback-controlled electro-burning in air and *I*-*V* characteristics were recorded for every device^39^. 2 μL of solutions of **MP** were drop-cast onto the nanogaps and the devices dried and measured.

### Determination of transport parameters

The transport measurements were performed in an OI-Triton dilution refrigerator using low-noise DC electronics (Delft- and home-made). The refrigerator is equipped with a vector magnet that can apply up to 1/6/1 T along the *B*_X_/*B*_Y_/*B*_Z_ axes. The electron temperature measured after the powder filtering is ~25 mK, corresponding to *k*_B_*T* ~2 µeV. The energies of the different molecular states were extracted from the transport data presented in Fig. 4 (field strength) and Fig. 6 (field angle) by the following procedure. Firstly, the *I*_SD_-*V*_G_ measurement taken at each *B*-field was differentiated with respect to *V*_G_. A Lorentzian function was fitted to each peak in the *dI*_SD_/*dV*_G_ vs. *V*_G_, and the centers of the Lorentzian are assigned to the potentials of the transitions. The potentials (in *V*_G_) are then converted into energies by multiplication by the gate lever arm, *β*. Thereby we can extract the energies of the ${{{{{{\bf{DyP}}}}}}}_{\uparrow \downarrow }^{+}$/ ${{{{{\bf{DyP}}}}}}$, and ${{{{{{\bf{DyP}}}}}}}_{\uparrow \uparrow }^{+}$/ ${{{{{\bf{DyP}}}}}}$ transitions. Simple subtraction of the energy of the ground state at *B* = 0 or at an angle of 0 yields the relative energies of ${{{{{{\bf{DyP}}}}}}}_{\uparrow \uparrow }^{+}$ and ${{{{{{\bf{DyP}}}}}}}_{\uparrow \downarrow }^{+}$ that is plotted as a function of *B*_Y_ (Fig. 4) and field angles (Fig. 6). The parameters extracted from the fits to the *E* vs. *B*_Y_ (e.g., *g*-factors) are reported in the main text with errors of ±2 standard deviations. The standard deviation is calculated from the variance returned from the fitting procedure. The fits of the nonlinear data in Fig. 6 are accompanied by standard errors of regression. A full list of the parameters of these fits is given in Supplementary Table 2. The method for estimating the current through AFM and FM channels is outlined in detail in the Supplementary Discussion.

### Cantilever-torque magnetometry

Cantilever-torque magnetometry (CTM) measures the magnetic torque that arises when a magnetic sample is placed in a homogeneous magnetic field, $B$. When $B$ is applied in a different direction than the magnetization $M$ there is a magnetic torque: $\zeta =M\times B$. The torque is a result of the energy dependence of the system as a function of the angle $\varepsilon$ between $M$ and $B$, $\zeta =-\partial E/\partial \varepsilon$. It can be shown that $\zeta$ can be expressed through orthogonal $\left(x,y,z\right)$ components:^40^

2 $${\zeta }_{y}={B}^{2}\left({M}_{x}/{B}_{x}-{M}_{z}/{B}_{z}\right){{\sin \varepsilon}} {\,}{{\cos \varepsilon}}$$

This result implies that ${\zeta }_{y}\,\ne \, 0$ only if ${M}_{x}/{B}_{x}-{M}_{z}/{B}_{z}={\chi }_{{xx}}-{\chi }_{{zz}}\,\ne \, 0$, i.e., if the system is magnetically anisotropic ($\chi$ is the magnetic susceptibility tensor). We note that $\zeta$ is a direct manifestation of the magnetic anisotropy in the sample and is insensitive to isotropic contributions to $M$.

CTM exploits magnetic torque by mounting a single-crystalline sample on a metallic cantilever before applying a magnetic field (see Supplementary Fig. 23 for device structure). Single crystals of **DyP** could not be obtained, due to the four trihexylsilyl groups and six dodecyl chains that decorate the structure (required for conferring solubility and increasing non-covalent interactions with the graphene electrodes respectively). Therefore CTM was carried out on **DyP’** (see Supplementary Fig. 23 for chemical structure) which retains the ligand-field environment of **DyP** but has substitutions to the molecular periphery to promote crystallization^26^. The direction of magnetization of the sample is pinned by anisotropy along a favorable crystallographic direction, and torque is generated by the misalignment $\varepsilon$ of the sample magnetization and applied field orientations. The torque bends the thin cantilever to reduce $\varepsilon$. In our setup the cantilever is separated from an underlying parallel metal plate by vacuum at a distance $d$. The small torque-induced deflections of the cantilever can be detected by measuring the capacitance between the cantilever and plate: $C\propto 1/d$. Finally, it can be shown that, $\zeta \left(B\right)={C}_{0}/C\,(B)-1$, where ${C}_{0}=C\,\left(B=0\right)$, which gives the qualitative $\zeta$-versus-$B$ behavior^40^.

We used CTM to assess qualitatively the magnetic anisotropy of the sample by measuring the torque response upon rotating ${{{{{\boldsymbol{B}}}}}}$ (with a fixed magnitude of 0.9 T) in the *xz* plane, as illustrated in Supplementary Fig. 23. The observed torque is a direct manifestation of magnetic anisotropy in this plane, and reveals information about “easy” and “hard” magnetic directions, which can be identified in the lab frame as $\phi \left(\zeta =0\right)$^41^. Furthermore, we have measured ${\zeta }_{y}$ as a function of ${{{{{\boldsymbol{B}}}}}}$ with fixed $\phi =-50^\circ$ and changing magnitude in order to detect magnetic hysteresis. From Eq. 2, $M\propto {\zeta }_{y}/B$, i.e., the anisotropic part of ${{{{{\boldsymbol{M}}}}}}$ in the chosen plane is given by ${\zeta }_{y}/B$. After several field-sweep cycles the ${\zeta }_{y}$ signal was averaged to yield the magnetic field curves as shown in Fig. 2b (main text). Note that because $\zeta$ vanishes at $B=0$ the calculation of ${\zeta }_{y}/B$ produces diverging values at fields close to zero. As a consequence, the signal close to *B* = *0* has been replaced by straight lines due to the vanishing zero-field torque signal. In our hysteresis measurements, we also observe instrumental noise around zero-field that adds to this effect.

### Ab initio calculations

In order to estimate the magnetic anisotropy in the **DyP** molecule, we have performed ab initio complete active space self-consistent field spin-orbit (CASSCF-SO) calculations with OpenMolcas^42^. We have used the geometry from X-ray crystallography without optimization and used basis sets from the ANO-RCC library^43–46^ with VTZP quality for Dy, VDZP quality for the atoms in the first coordination sphere, and VDZ quality for all other atoms. Two-electron integrals were decomposed using the Cholesky method with a 10^−8^ threshold to save resources. Relativistic effects were treated using the second-order Douglas–Kroll–Hess transformation where scalar contributions were taken into account at the spin-free level, and the spin-orbit coupling was applied in a state-interaction framework. The active space consisted of nine electrons in the seven 4 f orbitals of Dy(III), and we performed state-average CASSCF calculations for 18 sextets accounting for the lowest-lying ^6^H and ^6^F terms. Then, all 18 sextets were mixed by spin-orbit coupling to yield the final eigenstates (Supplementary Table 1).

## Supplementary information

Supplementary Information

## Data Availability

The datasets generated during and/or analyzed during the current study are included in this published paper (and its supplementary information files) or available from the corresponding author on request.
