# Supplementary material for: Exchange-induced spin polarization in a single magnetic molecule junction
Source: Nat Commun. 2022 Aug 3;13:4506. doi: 10.1038/s41467-022-31909-w (PMC9349289; doi:10.1038/s41467-022-31909-w)
Supplement: Supplementary file 1 — Supplementary Information [file 41467_2022_31909_MOESM1_ESM.pdf]

Supplementary Information:

Exchange-induced spin polarization in a single magnetic molecule junction

Tian Pei[1]†, James O. Thomas[1,2]†, Simen Sopp[1], Ming-Yee Tsang[1], Nicola Dotti[1], Jonathan Baugh[3], Nicholas F. Chilton[4], Salvador Cardona-Serra[5], Alejandro Gaita-Ariño[5], Harry L. Anderson[2], and Lapo Bogani[1]*

[1] Department of Materials, University of Oxford, 16 Parks Road, Oxford, OX1 3PH, UK.

[2] Department of Chemistry, University of Oxford, Chemistry Research Laboratory, Oxford, OX1 3TA, UK.

[3] Institute for Quantum Computing, University of Waterloo, 200 University Ave., N2L 3G1, Waterloo, ON, Canada.

[4] Department of Chemistry, School of Natural Sciences, University of Manchester, Oxford Road, Manchester, M13 9PL, UK.

[5] Instituto de Ciencia Molecular, Universidad de València, 2 C/Catedrático José Beltrán, Paterna, Valencia, Spain.

* Correspondence to: lapo.bogani@materials.ox.ac.uk

† These authors contributed equally to this work.

Contents

Supplementary Methods 3

- General Synthetic Details 3
- Synthetic Protocols 3
- Supporting Characterization Data 6
- Torque Magnetometry Setup 17

Supplementary Discussion 18

- Additional Stability Diagrams 18
- Determination of Molecule-Electrode Couplings 19
- Assignment of Molecular Oxidation States 20
- Transport Simulation 22
- Spin Polarization 24

Supplementary Tables 26

Supplementary References 27

Supplementary Methods

General Synthetic Details

Reagents were purchased from commercial sources. Solvents were used as supplied (analytical/HPLC-grade from Fisher or Sigma-Aldrich) or if dry solvents were required, taken from a solvent drying system (MBraun MB-SPS-5-Bench Top) under nitrogen. Petrol ether (PE) over a boiling point range of 40–60 °C was used. Eluent mixtures are reported in volume: volume. Column chromatography was carried out using Merck Geduran silica gel 60 under N_2 pressure. TLC was carried out on Merck silica gel 60 F254 Al plates. MALDI-TOF-MS was carried out in positive reflectron mode using a Bruker MALDI microflex instrument with dithranol as a matrix. NMR spectroscopy measurements were recorded using a Bruker AVII400 instrument. All peaks were referenced to the residual solvent peak. UV-vis spectra were recorded using a PerkinElmer Lambda 25 instrument. Size exclusion chromatography (SEC) was carried out using Bio-Beads S-X1, 200–400 mesh (Bio Rad). Recycling gel permeation chromatography (GPC) was carried out on Shimadzu system equipped with a set of JAIGEL 3H (20 × 600 mm) and JAIGEL 4H (20 × 600 mm) columns with a flow rate of 3.5 mL/min. Analytical GPC was performed on a JAIGEL H-P pre-column, a JAIGEL 3H-A (8 mm × 500 mm) and a JAIGEL 4H-A column (8 mm × 500 mm) in series with tetrahydrofuran/1% pyridine as eluent.

Synthetic Protocols

The synthesis of the free-base porphyrin (FBP), the capping ligand (NaL_{OEt}), and the anchor group 1,3,6-*tris*(dodecyloxy)-8-ethynylpyrene (TDP) have been reported previously.[1, 2, 3]

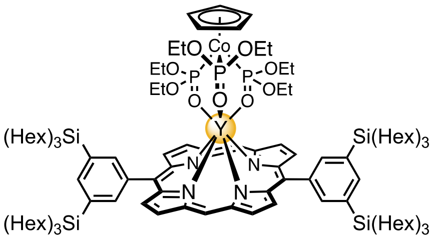

Synthesis of H_2YP: The free-base porphyrin FBP (95 mg, 60 µmol), $YCl_3.6H_2O$ (116 mg, 0.60 mmol), benzimidazole (1.0 g), imidazole (2.0 g) and diphenyl ether (2.0 g) were added to a dry Schlenk tube. The reaction mixture was heated to 200 °C overnight while stirring under argon. The mixture was allowed to cool and $CHCl_3$ (30 mL) was added. The solution was washed with water (3 × 30 mL), filtered over $MgSO_4$ and dried. The diphenyl ether was removed under high vacuum using a Hickmann distillation head. The crude product was stirred in $CHCl_3$ (15 mL) with NaL_{OEt} (300 mg, 0.30 mmol) for 1 hour. A silica column was used to purify the product, with a solvent gradient (100:0 to 6:1 PE:ethylacetate). The solvent was removed under reduced pressure to yield H_2YP as a pink solid (22 mg, 17 %).

^{1}H NMR (400 MHz, Chloroform-d) δ_H / ppm: 10.23 (s, 2H), 9.21 (d, *J* = 4.3 Hz, 4H), 8.87 (d, *J* = 4.3 Hz, 4H), 8.67 (t, *J* = 1.5 Hz, 2H), 7.94 (d, *J* = 1.2 Hz, 2H), 7.83 (t, *J* = 1.4 Hz, 2H), 4.05 (s, 5H), 2.55 – 2.36 (m, 12H), 1.67 – 1.54 (m, 12H), 1.53 – 0.70 (m, 162H)

MALDI-TOF m/z: 2214.4 ($C_{121}H_{207}CoN_4O_9P_3Si_4Y$, M^+ requires 2214.3)

UV/vis ($CHCl_3$, 298 K) λ_{max} / nm (log ε): 418 (5.56), 546 (4.18).

Synthesis of Br_2YP: A solution of freshly recrystallized *N*-bromosuccinimide (NBS, 3.5 mg, 20 µmol) in dichloromethane (1.5 mL) was added dropwise to a solution of the porphyrin H_2YP (20 mg, 9.0 µmol) in dichloromethane (3 mL) with stirring. The reaction was monitored by TLC, and after completion the mixture was passed over a silica plug, eluting with PE:CH_2Cl_2, 3:1. The solvent was removed to yield the product as a purple solid (21 mg, 100%).

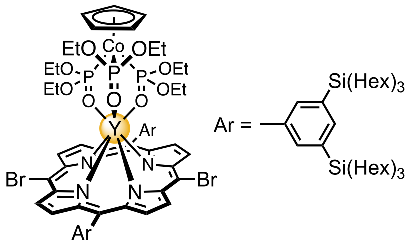

^1H NMR (400 MHz, Chloroform-d) δ_H / ppm: 9.53 (d, *J* = 4.6 Hz, 4H), 8.66 (d, *J* = 4.6 Hz, 4H), 8.56 (t, *J* = 1.5 Hz, 2H), 7.94 (d, *J* = 1.2 Hz, 2H), 7.72 (t, *J* = 1.5 Hz, 2H), 4.12 (s, 5H), 2.66 – 2.55 (m, 12H), 1.63 – 0.73 (m, 174H)

MALDI-TOF m/z: 2373.5 ($C_{121}H_{205}Br_2CoN_4O_9P_3Si_4Y$, M^+ requires 2373.1)

UV/vis (CHCl3, 298 K) λ_{max} / nm (log ε): 429 (5.48), 436 (5.51), 568 (4.20), 608 (3.99).

Synthesis of YP: The bromoporphyrin Br_2YP (20 mg, 8.4 µmol) was placed in a dry Schlenk tube along with $Pd(PPh_3)_4$ (2.0 mg, 1.7 µmol), CuI (0.2 mg, 0.9 µmol) and 1,3,6-*tris*(dodecyloxy)-8-ethynylpyrene (26 mg, 34 µmol) under argon. Dry THF (0.8 mL) and dry di-*iso*-propylamine (0.8 mL) were added and the mixture was immediately frozen, and subjected to three freeze-pump-thaw cycles. After 16 hours at 65 °C the reaction mixture was passed over a short plug in PE:CH_2Cl_2 (3:1). The mixture was passed over a silica column using an eluent gradient from 100:1 to 6:1 PE:ethylacetate. The mixture was further purified on a SEC column, eluting with toluene. Recycling GPC using toluene:pyridine 99:1 was used as a final purification step. The solvent removed under high vacuum to yield the final product YP as a brown solid (2.4 mg, 8 %).

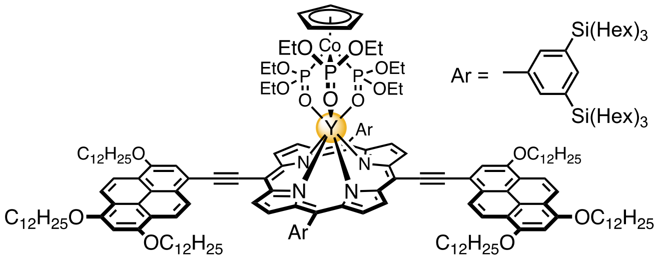

^1H NMR (400 MHz, Chloroform-d) δ_H 9.80 (d, *J* = 4.5 Hz, 4H), 8.91 (d, *J* = 9.3 Hz, 2H), 8.72 (d, *J* = 4.3 Hz, 4H), 8.62 (s, 2H), 8.43 (d, *J* = 3.1 Hz, 2H), 8.40 (d, *J* = 3.1 Hz, 2H), 8.32 (d, *J* = 9.3 Hz, 2H), 8.08 (s, 2H), 7.95 (s, 2H), 7.80 (s, 2H), 7.18 (s, 2H), 4.48 (t, *J* = 6.4 Hz, 4H), 4.35 (t, *J* = 6.5 Hz, 8H), 4.14 (s, 5H), 2.77 – 2.69 (m, 12H), 2.07 (m, 12H), 1.75 – 0.70 (m, 312H)

MALDI-TOF m/z: 3769.4 ($C_{229}H_{367}CoN_4O_{15}P_3Si_4Y$, M^+ requires 3768.5)

UV/vis (toluene, 298 K) λ_{max} / nm (log ε): 384 (4.61) 478 (5.08), 700 (4.79).

Synthesis of H_2DyP: The free-base porphyrin FBP (85 mg, 53 µmol), $DyCl_3.6H_2O$ (189 mg, 0.50 mmol), benzimidazole (1.0 g), imidazole (1.2 g) and diphenyl ether (1.8 g) were added to a dry Schlenk tube. The reaction mixture was heated to 210 °C overnight while stirring under argon. The mixture was allowed to cool and $CHCl_3$ (30 mL) was added. The solution was washed with water (3 × 50 mL), filtered over $MgSO_4$ and dried. The diphenyl ether was removed under high vacuum using a Hickmann distillation head. The crude product was stirred in $CHCl_3$ (15 mL) with NaL_{OEt} (65 mg, 65 µmol) for 1 hour. A silica column was used to purify the product, with a solvent gradient (100:0 to 6:1 PE:ethylacetate). The solvent was removed under reduced pressure to yield H_2DyP as a pink solid (85 mg, 70 %).

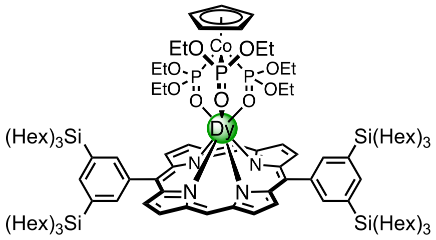

MALDI-TOF m/z: 2288.6 ($C_{121}H_{207}CoDyN_4O_9P_3Si_4$, M^+ requires 2288.3)

UV/vis ($CHCl_3$, 298 K) λ_{max} / nm (log ε): 419 (5.50), 547 (4.13).

Synthesis of Br_2DyP: A solution of freshly recrystallized *N*-bromosuccinimide (NBS, 11 mg, 60 µmol) in dichloromethane (2 mL) was added dropwise to a solution of the porphyrin H_2DyP (62 mg, 27 µmol) in dichloromethane (3 mL) with stirring. The reaction was monitored by TLC, and after completion the mixture was passed over a silica plug, eluted with PE:CH_2Cl_2, 3:1. The solvent was removed to yield Br_2DyP as a purple solid (56 mg, 85%).

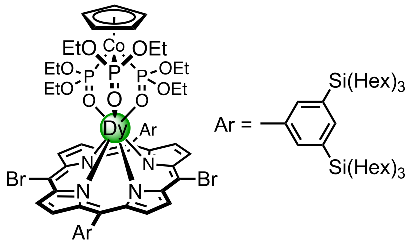

MALDI-TOF m/z: 2446.5 ($C_{121}H_{205}Br_2CoDyN_4O_9P_3Si_4$, M^+ requires 2446.1)

UV/vis (CHCl3, 298 K) λ_{max} / nm (log ε): 429 (5.51), 436 (5.54), 568 (4.23), 608 (3.98).

Synthesis of DyP: The bromoporphyrin Br_2DyP (20 mg, 8.3 µmol) was placed in a dry Schlenk tube along with $Pd(PPh_3)_4$ (2.0 mg, 1.7 µmol), CuI (0.5 mg, 2.5 µmol) and 1,3,6-*tris*(dodecyloxy)-8-ethynylpyrene (19.2 mg, 25 µmol) under argon. Dry THF (0.8 mL) and dry di-*iso*-propylamine (0.8 mL) were added and the mixture was immediately frozen, and subjected to three freeze-pump-thaw cycles. After 2 hours at 50 °C the reaction mixture was passed over a short plug in PE:CH_2Cl_2:PE (3:1). The mixture was passed over a silica column using an eluent gradient from 100:1 to 6:1 PE:ethylacetate. The mixture was further purified on a SEC column, eluting with toluene. Recycling GPC using toluene:pyridine 99:1 was used as a final purification step. The solvent was removed to yield the final product DyP as a brown solid (4.4 mg, 14 %).

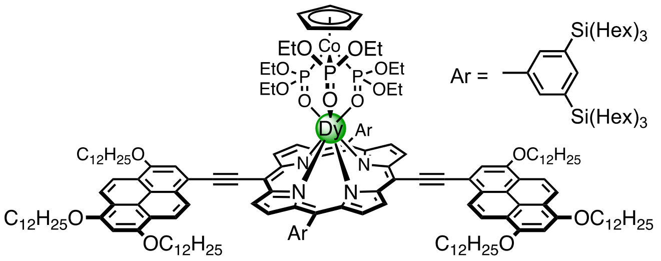

MALDI-TOF m/z: 3843.1 ($C_{229}H_{367}CoDyN_4O_{15}P_3Si_4$ M^+ requires 3842.5)

UV/vis (toluene, 298 K) λ_{max} / nm (log ε): 383 (4.69) 478 (5.17), 701 (4.95).

Supporting Characterization Data

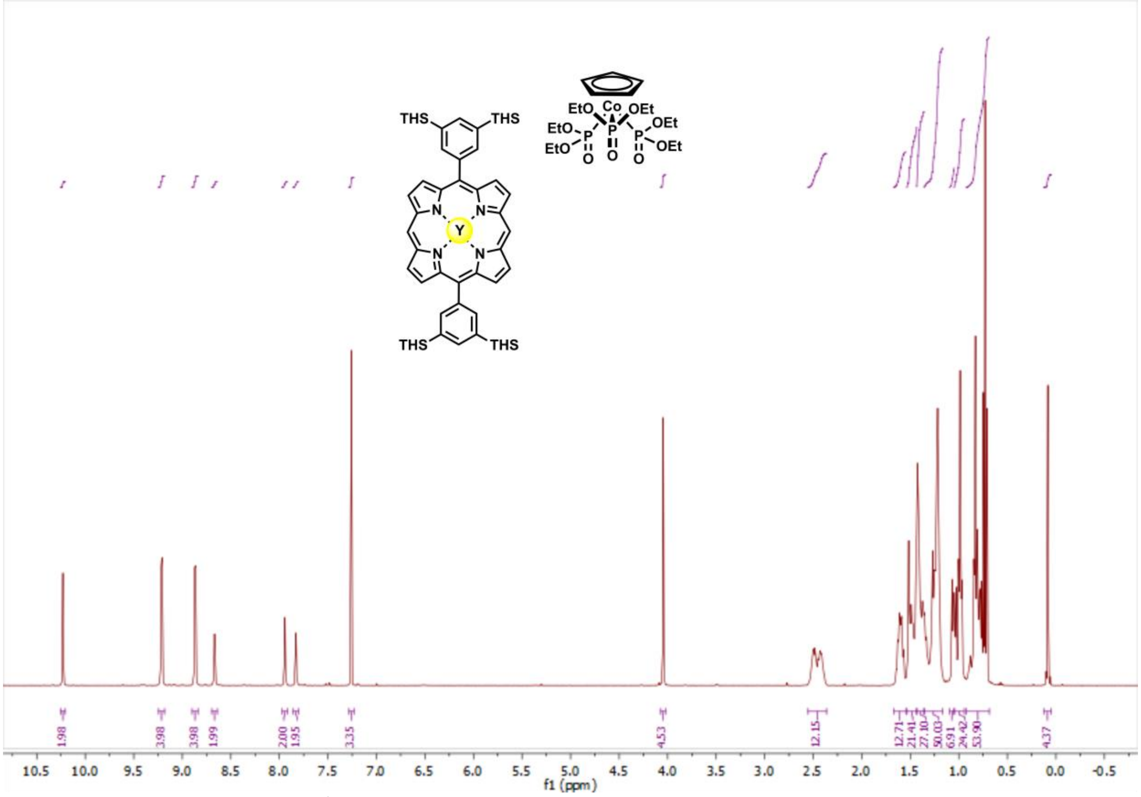

Supplementary Figure 1. ^{1}H NMR spectrum (400 MHz, chloroform-d, 298 K) of H_2YP.

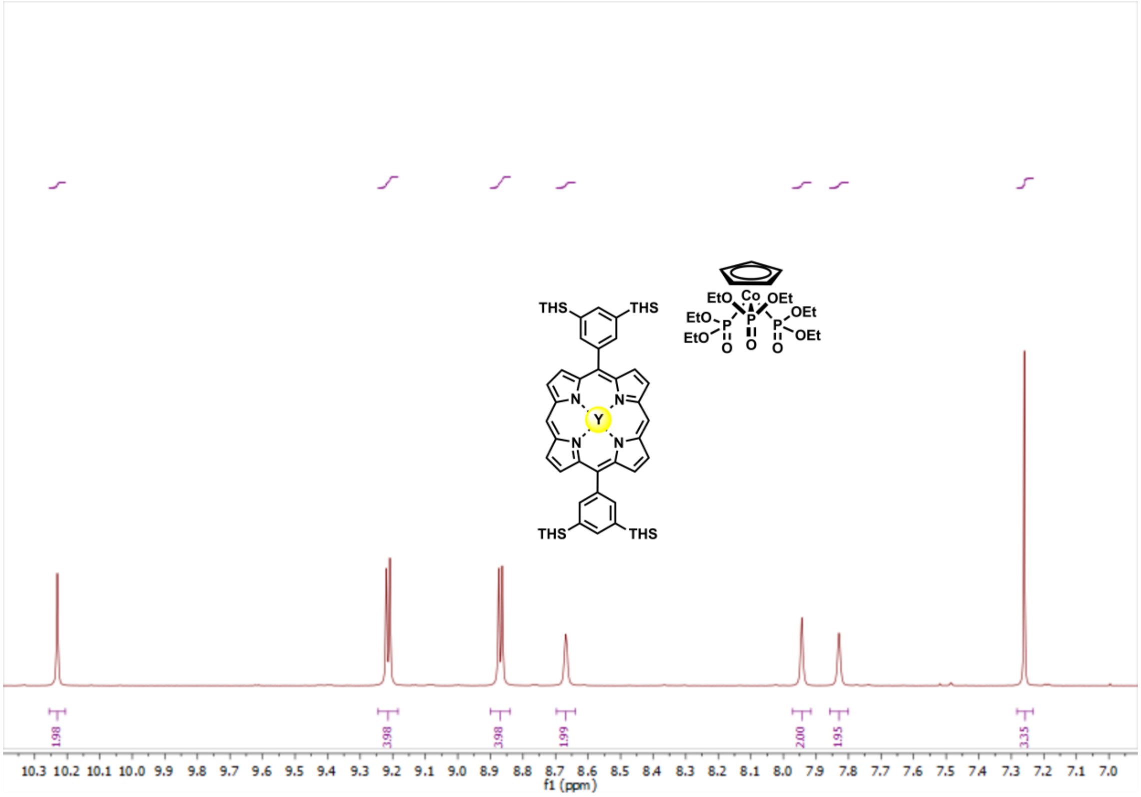

Supplementary Figure 2. ^{1}H NMR spectrum (400 MHz, chloroform-d, 298 K) of H_2YP, showing the downfield region.

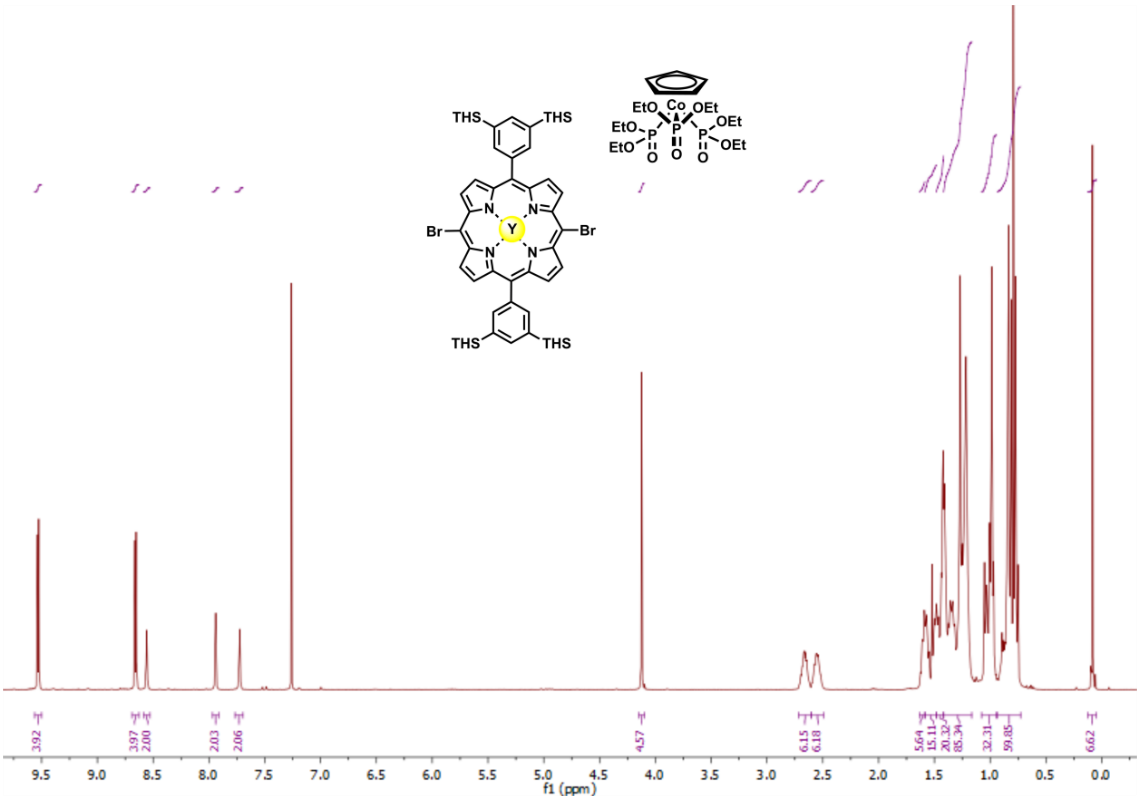

Supplementary Figure 3. ^{1}H NMR spectrum (400 MHz, chloroform-d, 298 K) of Br_2YP.

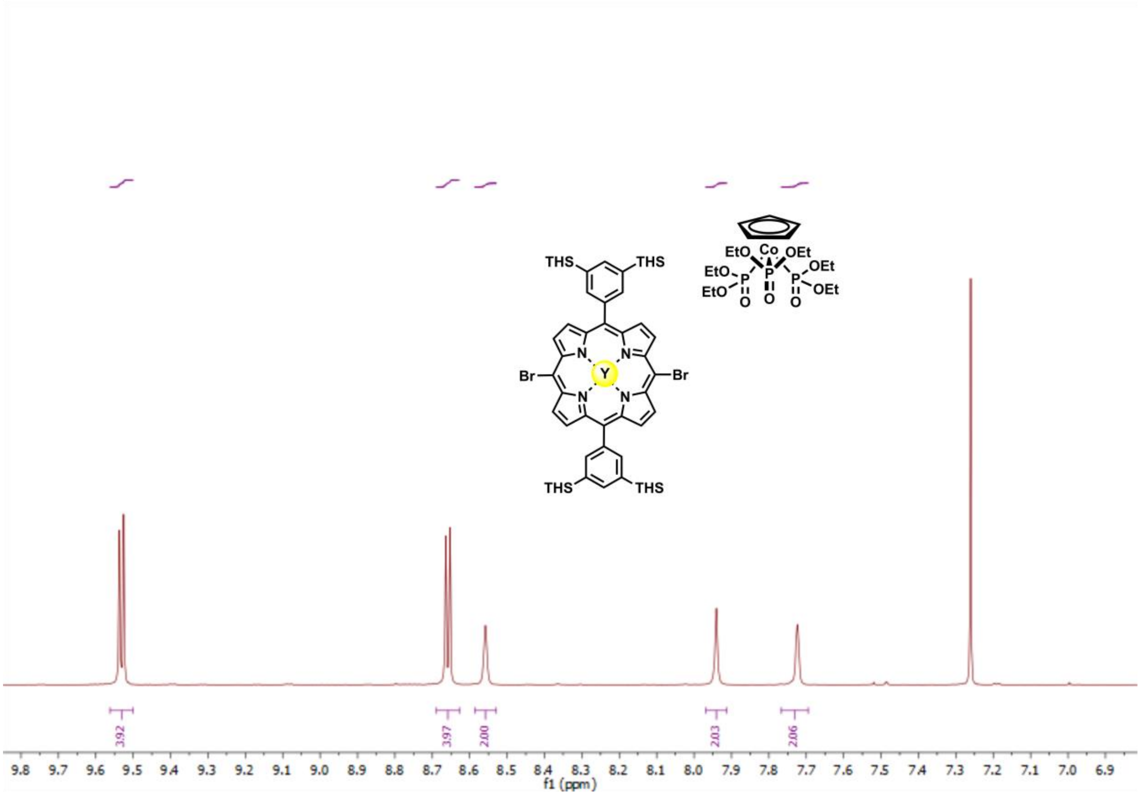

Supplementary Figure 4. ^{1}H NMR spectrum (400 MHz, chloroform-d, 298 K) of Br_2YP, showing downfield portion only.

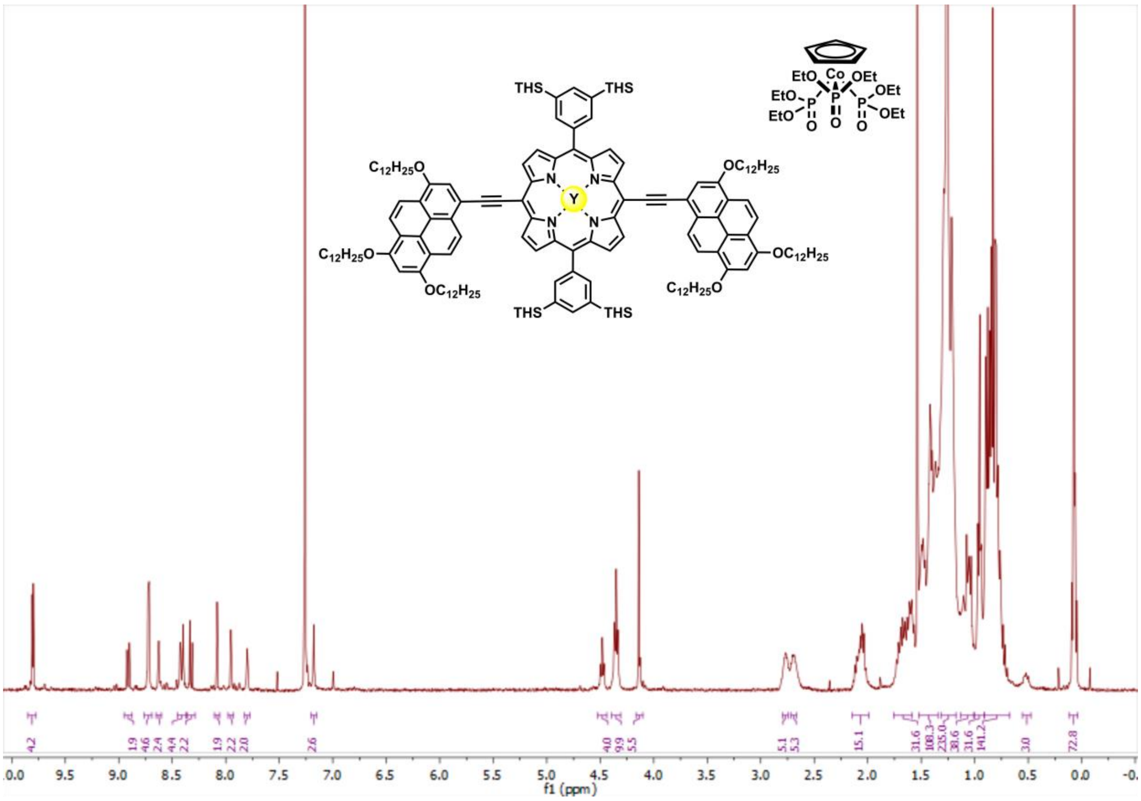

Supplementary Figure 5. ^{1}H NMR spectrum (400 MHz, chloroform-d, 298 K) of YP.

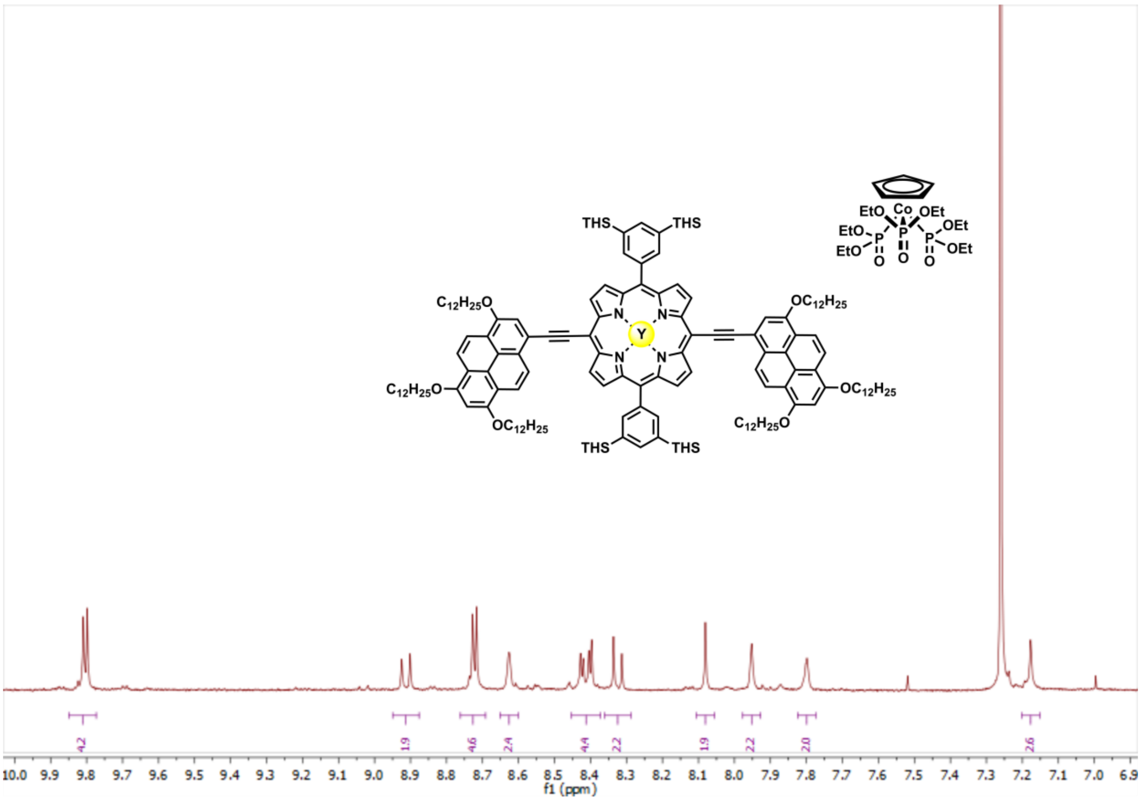

Supplementary Figure 6. ^{1}H NMR spectrum (400 MHz, chloroform-d, 298 K) of YP, showing downfield portion only.

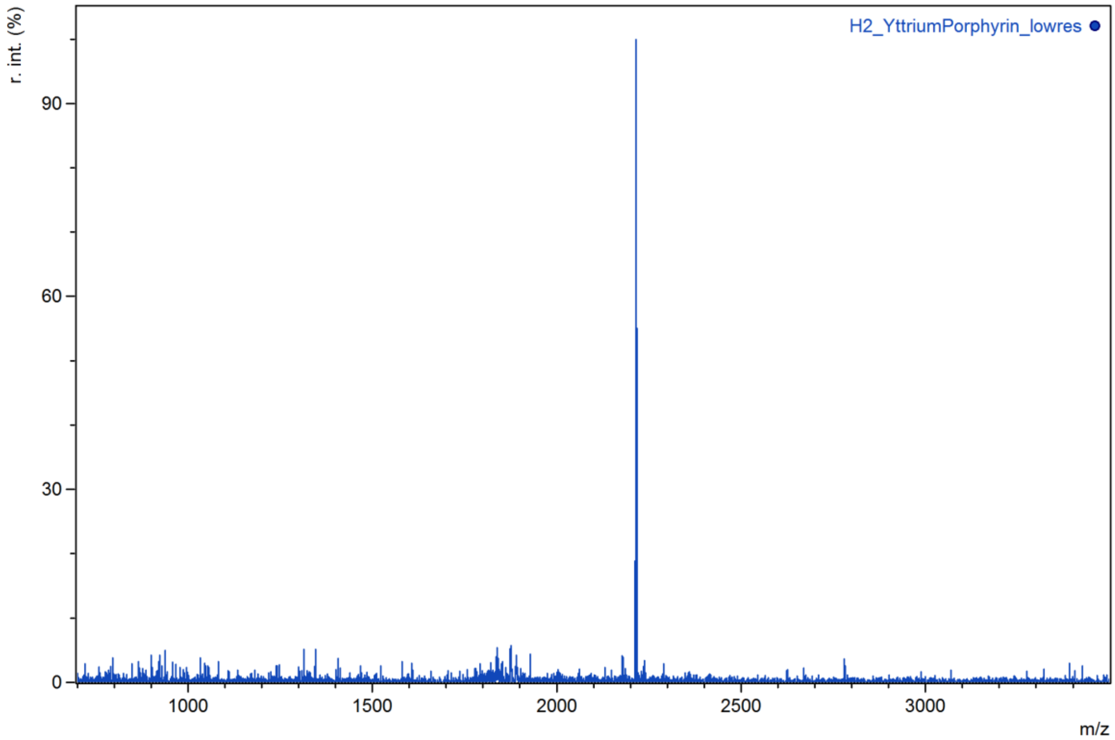

Supplementary Figure 7. MALDI-TOF mass spectrum of H_2YP.

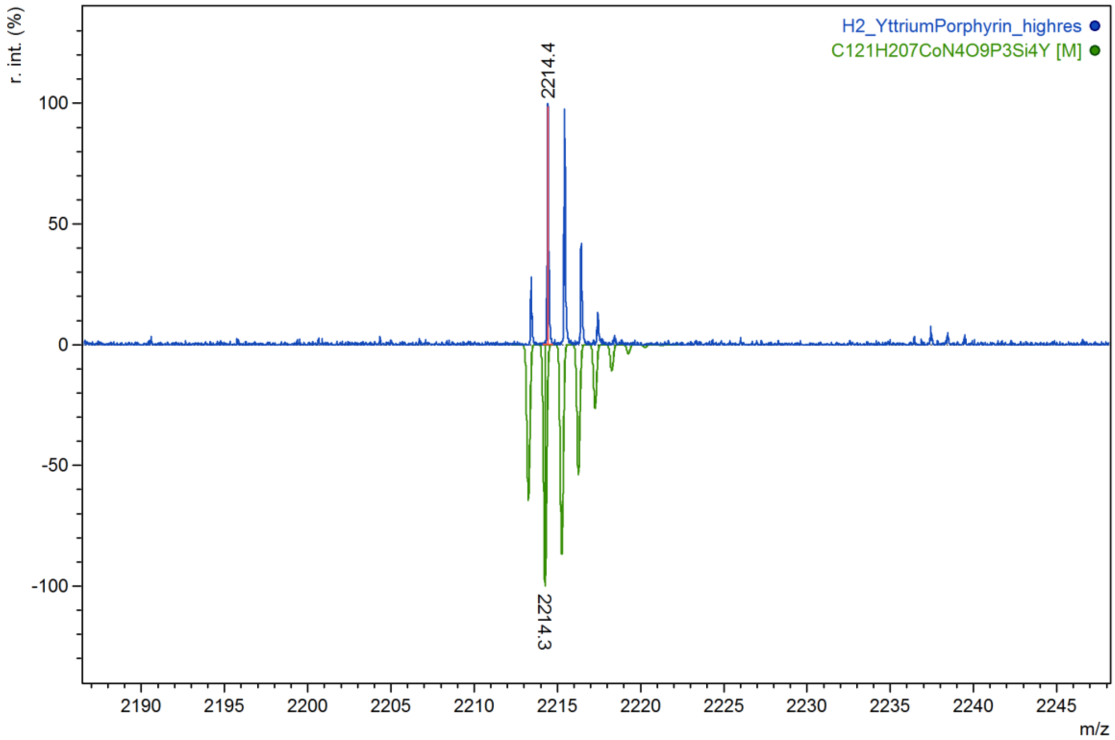

Supplementary Figure 8. MALDI-TOF mass spectrum of H_2YP (blue), compared to calculated isotope pattern (green).

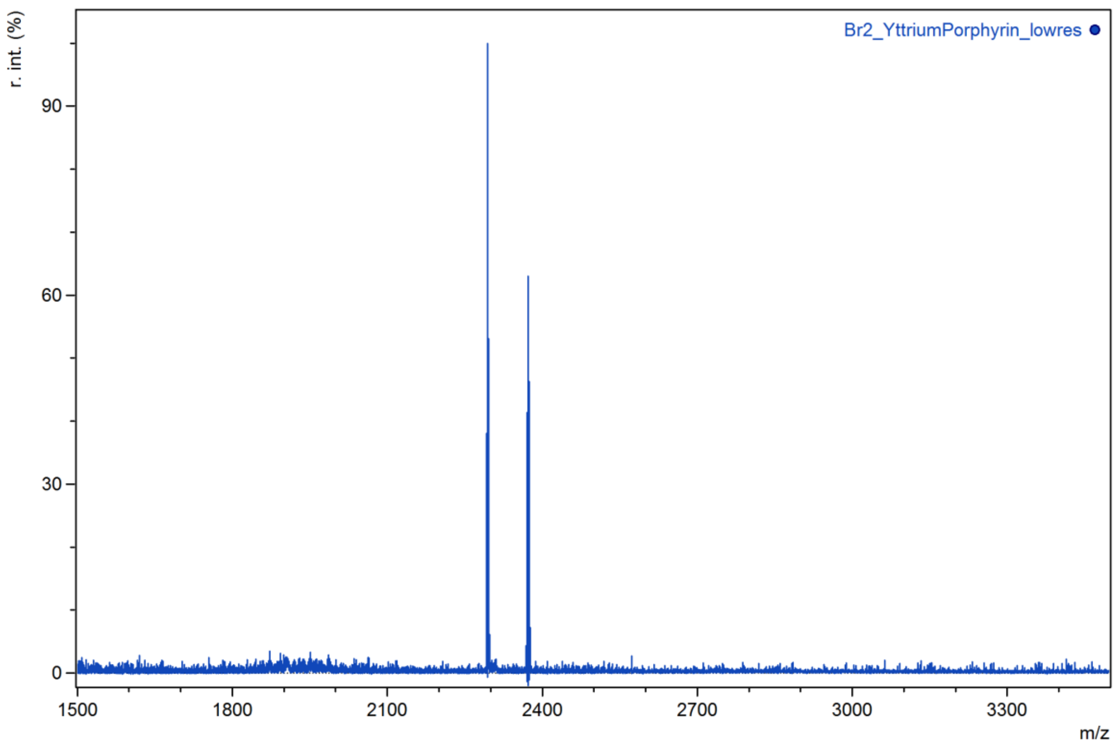

Supplementary Figure 9. MALDI-TOF mass spectrum of Br_2YP. There are two peaks as Br_2YP loses a bromine upon irradiation/ionization.

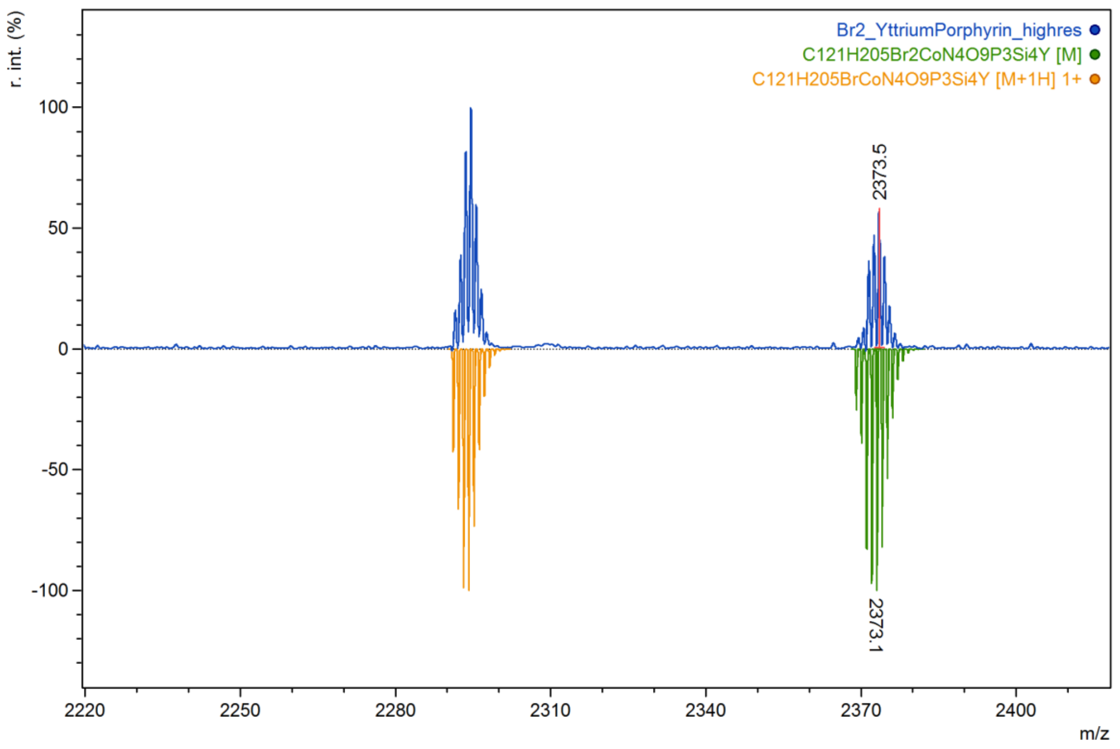

Supplementary Figure 10. MALDI-TOF mass spectrum of Br_2YP (blue), compared to calculated isotope pattern of molecular ion (green), and molecular ion minus bromine (orange).

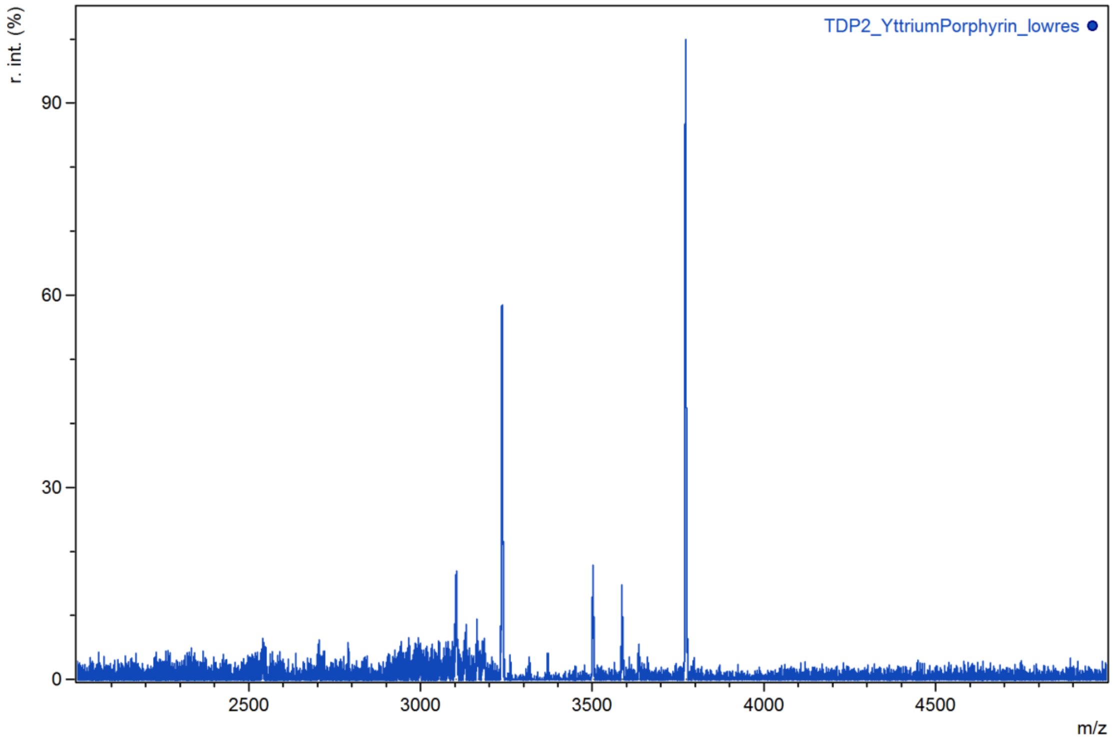

Supplementary Figure 11. MALDI-TOF mass spectrum of YP, the second largest peak is the mass of the molecular ion minus the capping group.

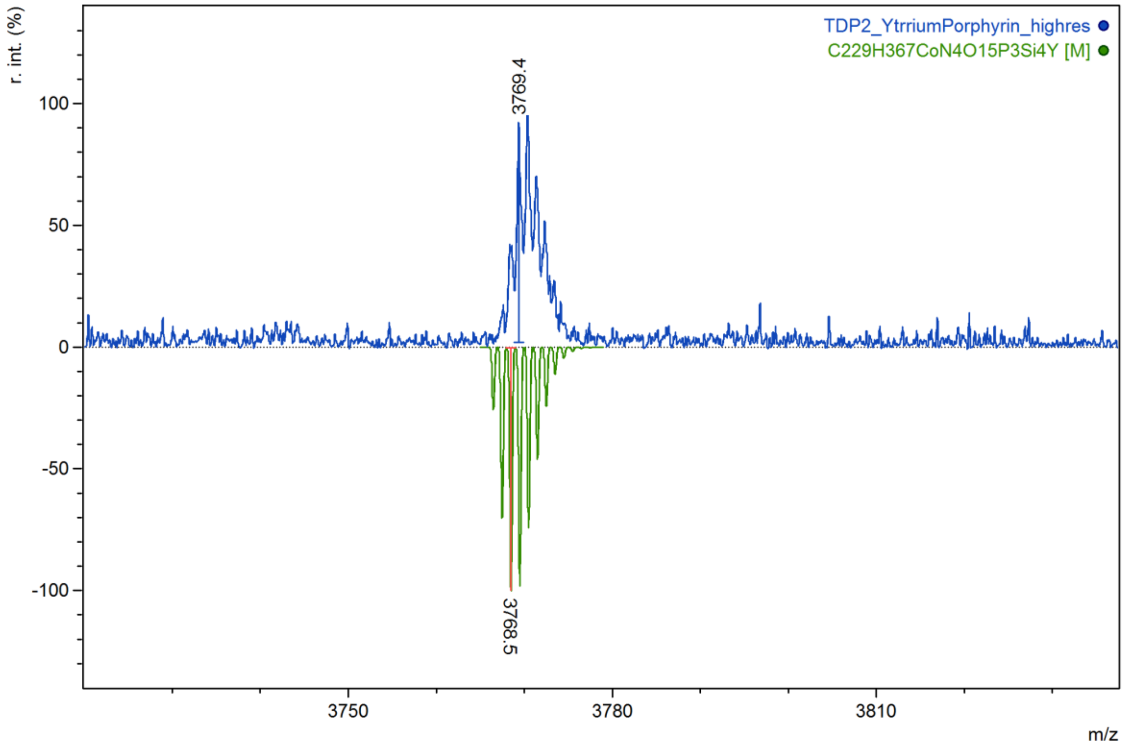

Supplementary Figure 12. MALDI-TOF mass spectrum of YP (blue), compared to the calculated isotope pattern (green).

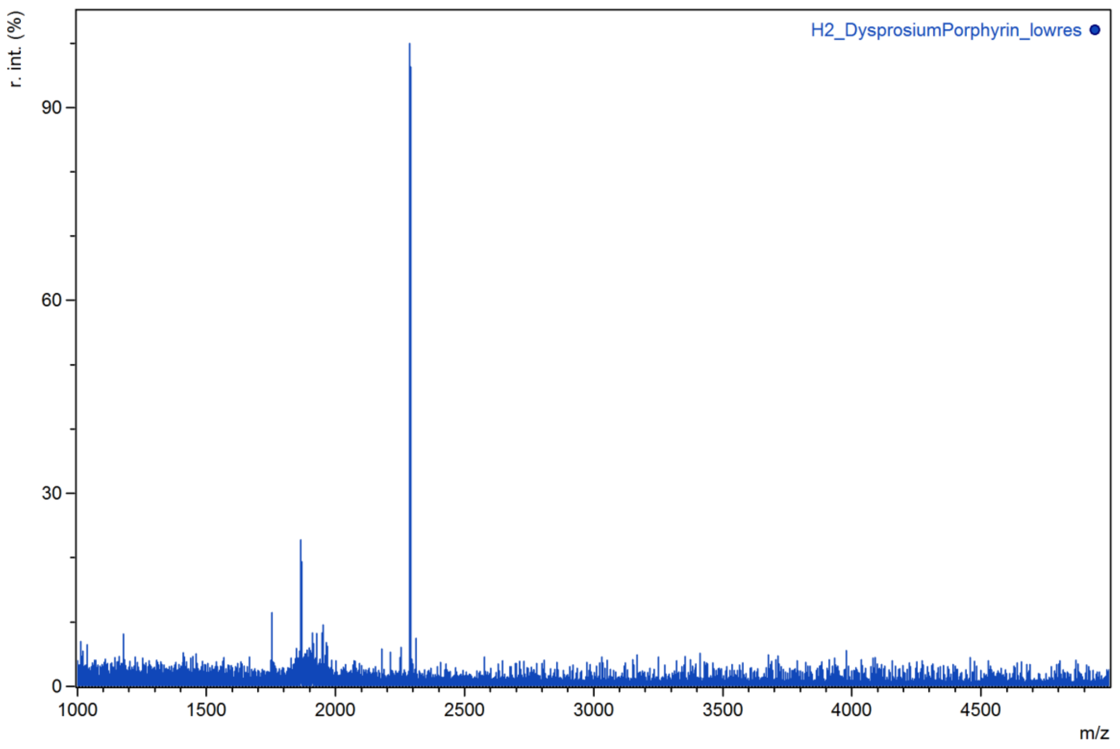

Supplementary Figure 13. MALDI-TOF mass spectrum of H_2DyP.

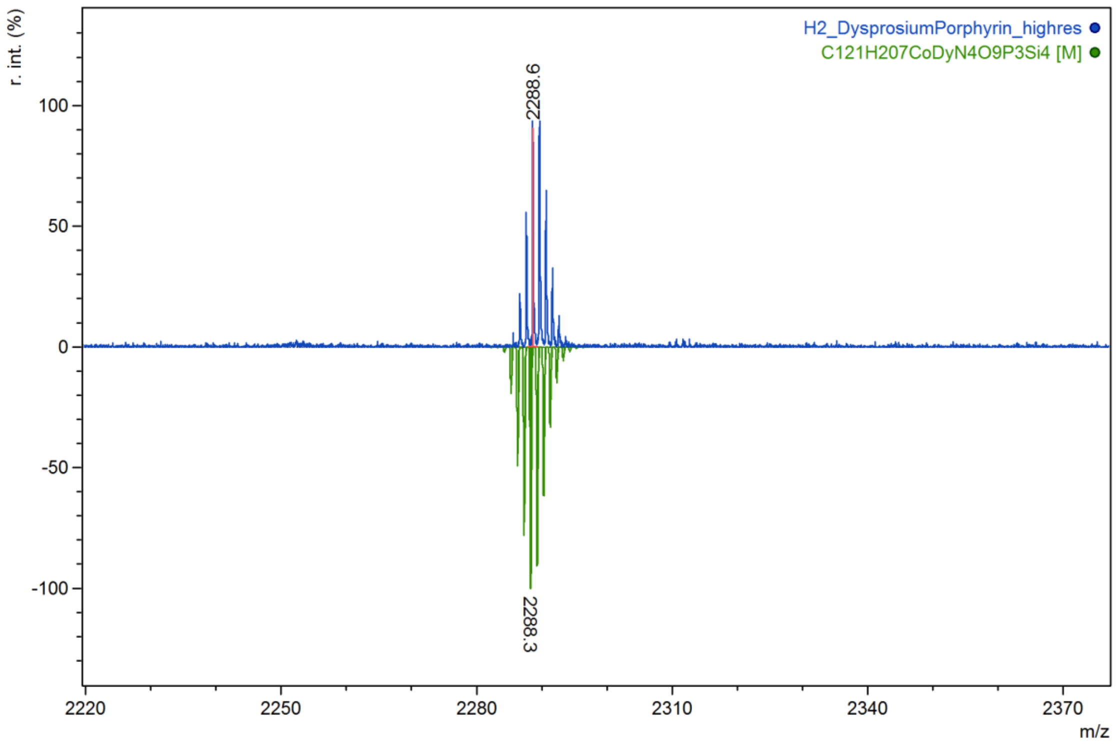

Supplementary Figure 14. MALDI-TOF mass spectrum of H_2DyP (blue), compared to calculated isotope pattern (green).

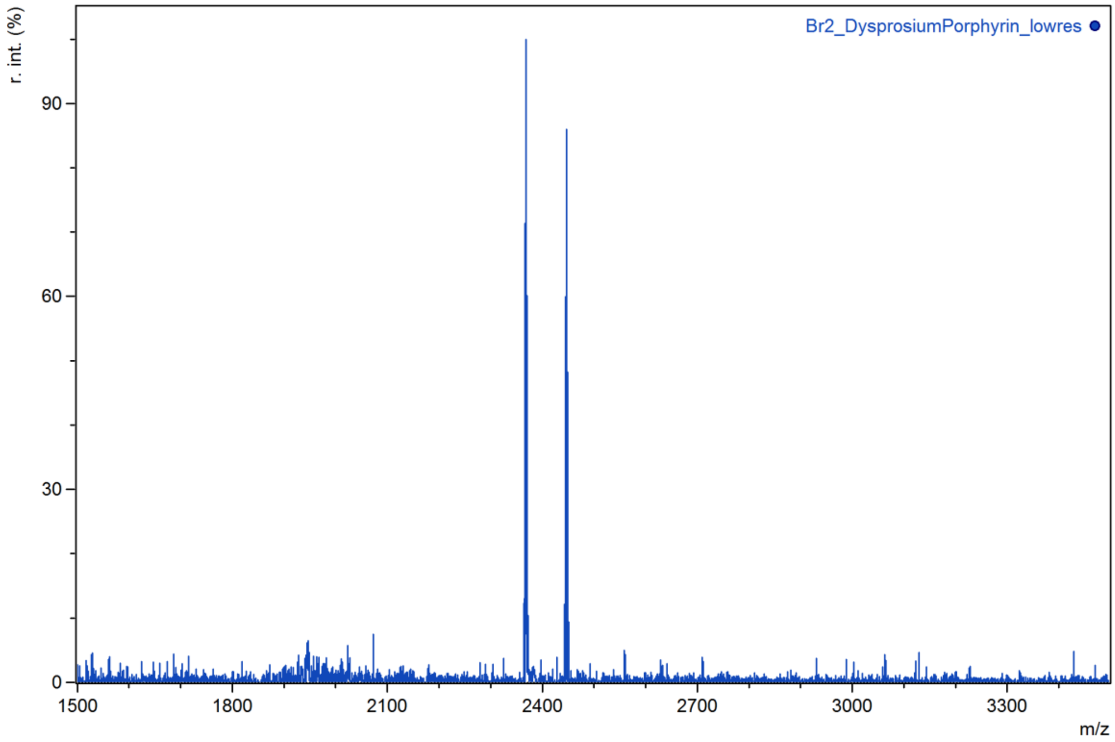

Supplementary Figure 15. MALDI-TOF mass spectrum of Br_2DyP. There are two peaks as Br_2DyP loses bromine upon irradiation/ionization.

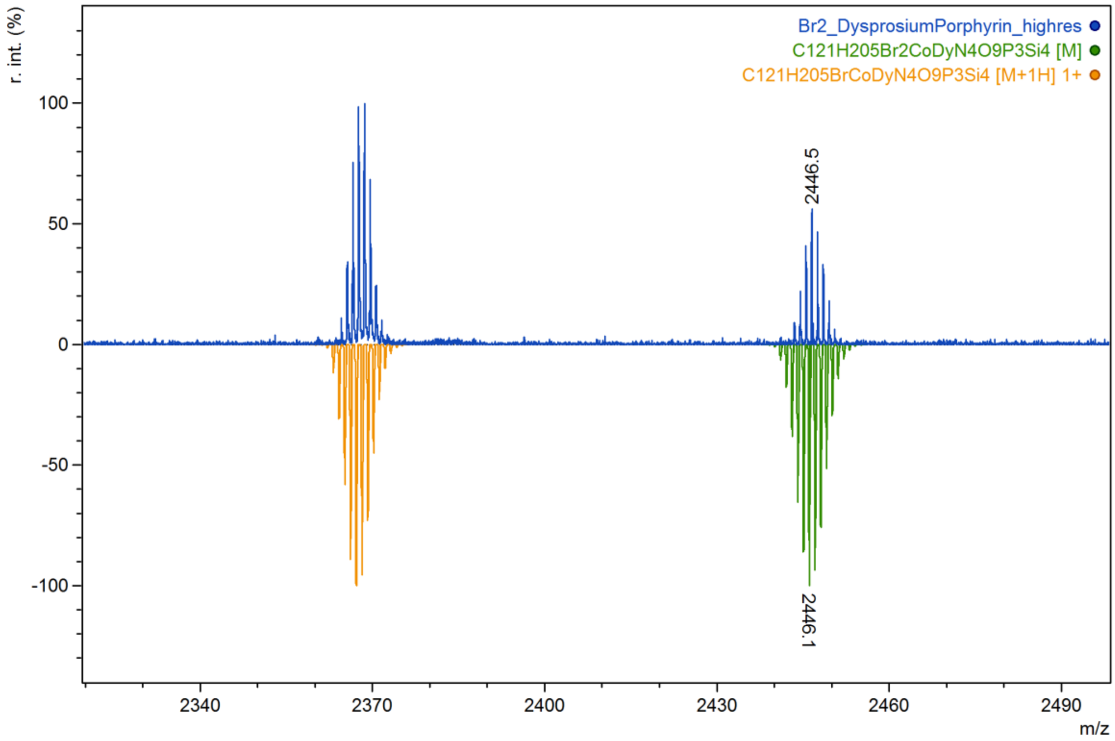

Supplementary Figure 16. MALDI-TOF mass spectrum of Br_2DyP (blue), compared to calculated isotope pattern of the molecular ion (green), and the molecular ion minus bromine (orange).

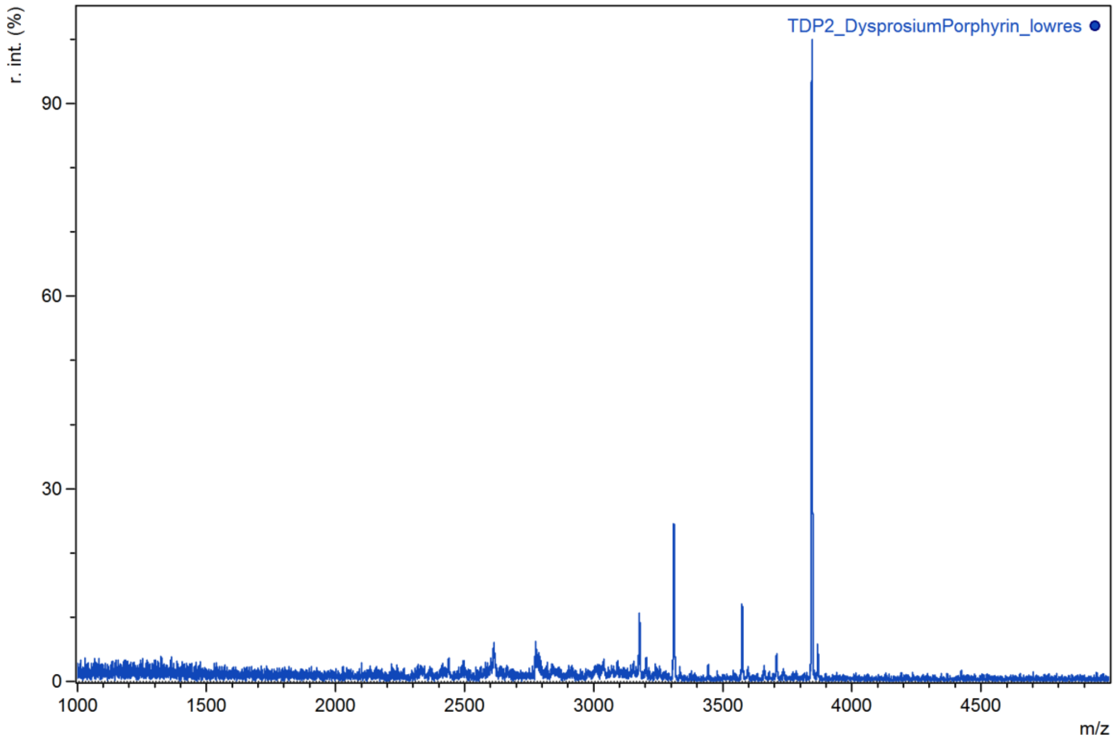

Supplementary Figure 17. MALDI-TOF mass spectrum of DyP, the second largest peak is the mass of the molecular ion minus the capping group.

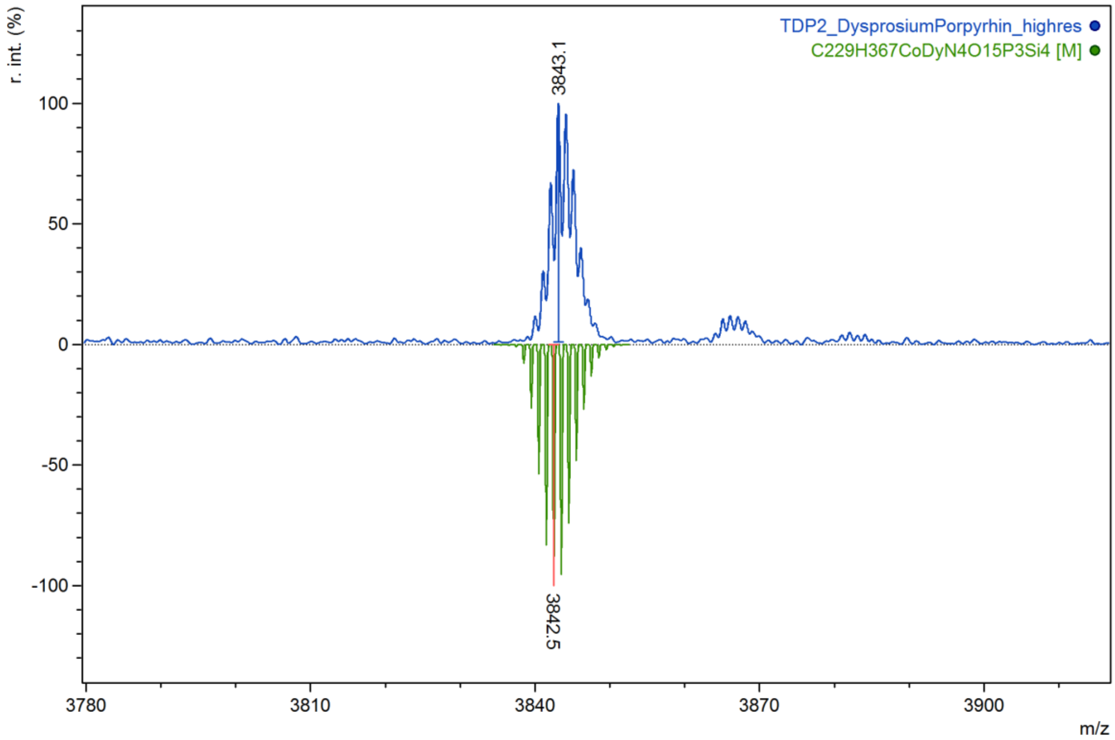

Supplementary Figure 18. MALDI-TOF mass spectrum of DyP (blue), compared to the calculated isotope pattern (green).

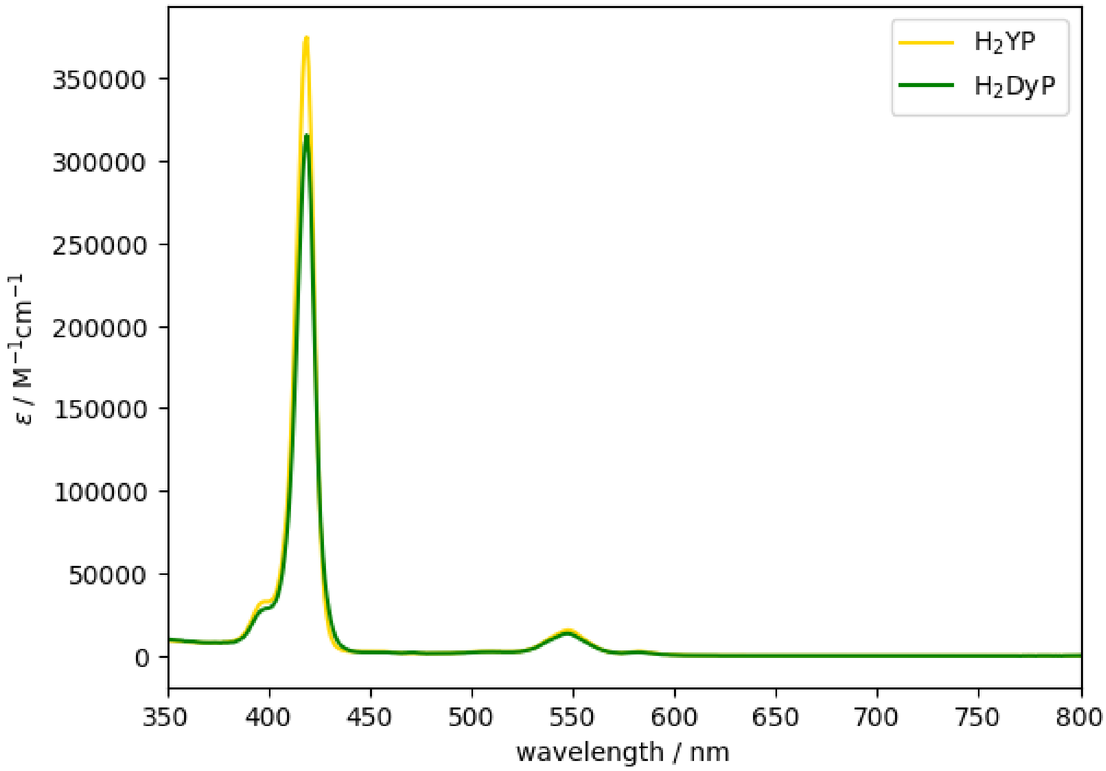

Supplementary Figure 19. UV/vis absorbance spectra ($CHCl_3$, 298 K) of H_2YP and H_2DyP.

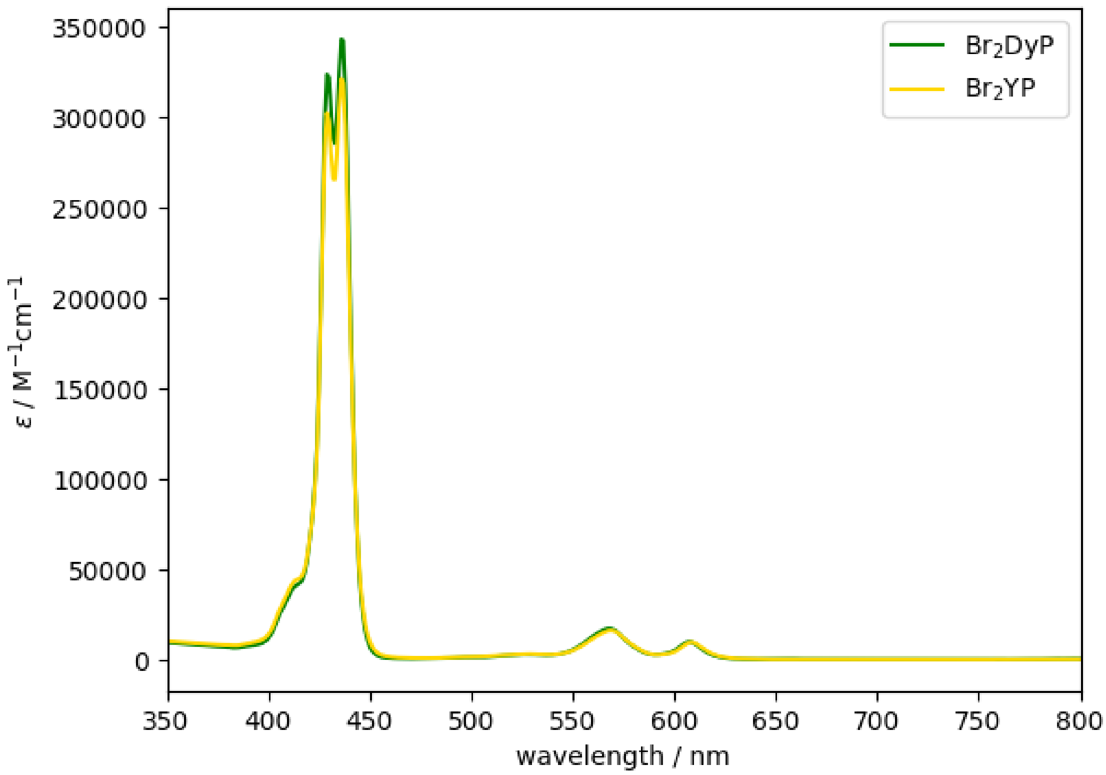

Supplementary Figure 20. UV/vis absorbance spectra ($CHCl_3$, 298 K) of Br_2YP and Br_2DyP.

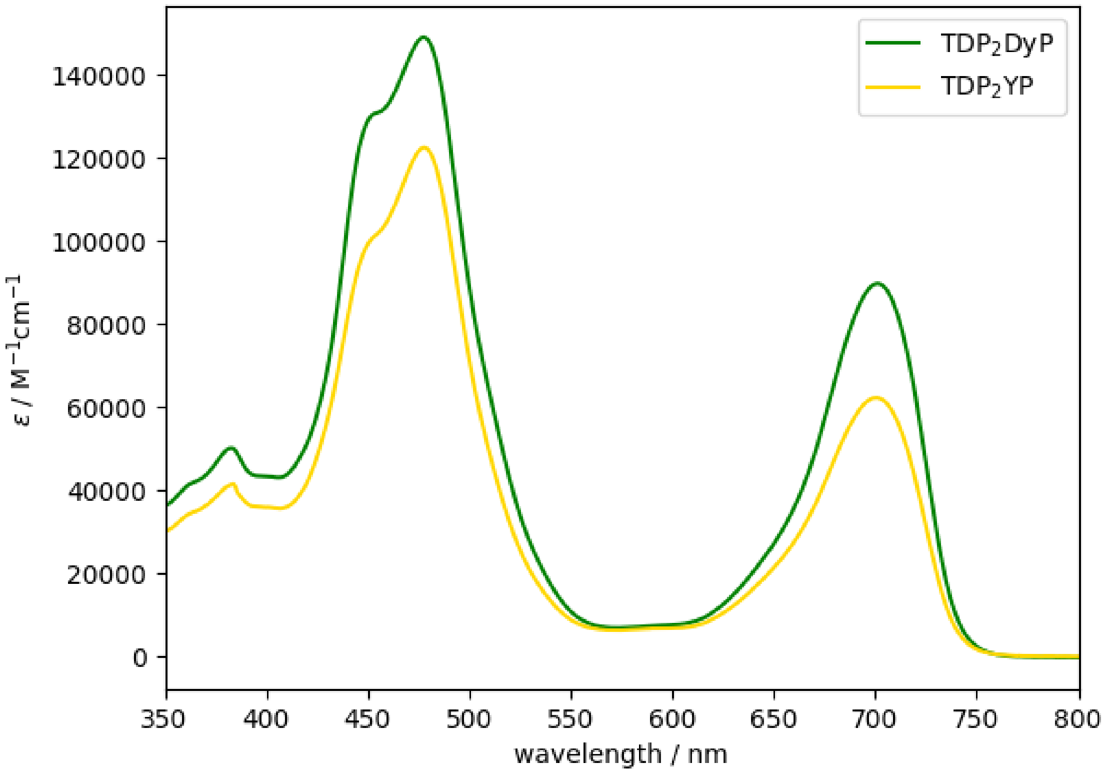

Supplementary Figure 21. UV/vis absorbance spectra (toluene, 298 K) of YP and DyP.

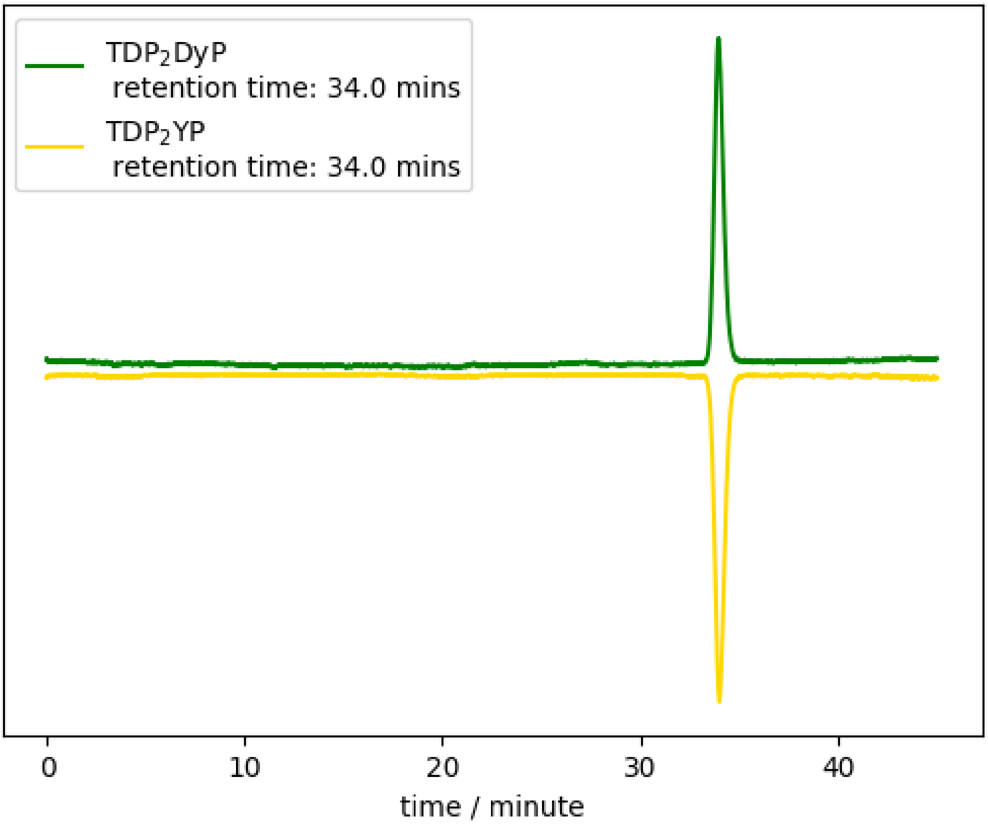

Supplementary Figure 22. GPC traces (1% pyridine in THF, λ = 480 nm) of YP and DyP. The YP trace has had its absorbance inverted so both traces can be clearly seen.

.

Torque Magnetometry Setup

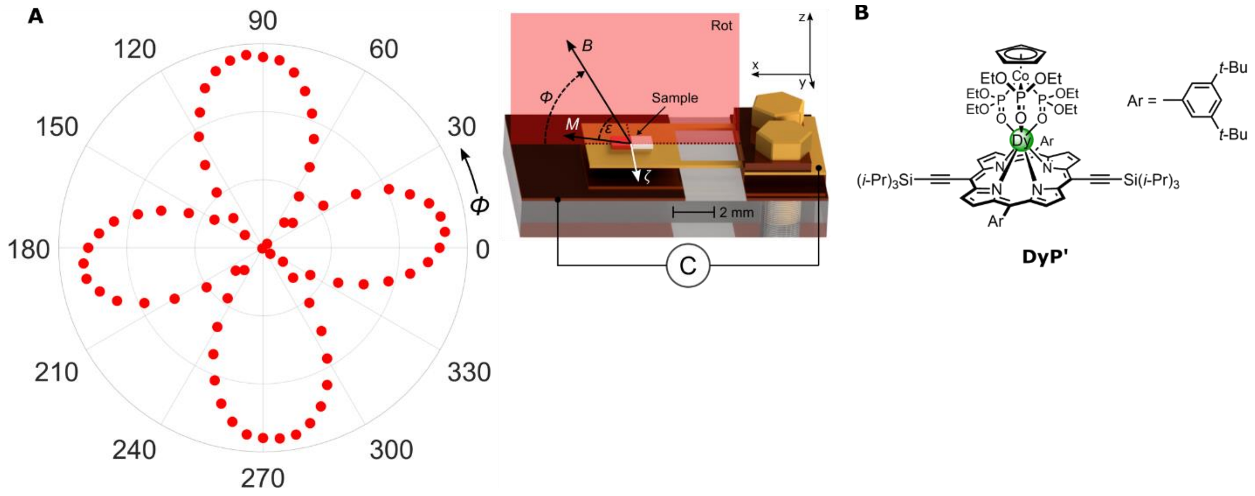

Supplementary Figure 23. Torque Magnetometry. (a) Angular dependence of the torque signal measured using CTM. The torque (red dots) is plotted radially in arbitrary units versus the angle ϕ of the applied field *B*. The inset shows the CTM setup with schematics for the rotation plane and capacitive detection. The single crystalline **DyP`** sample was mounted centrally on the copper beryllium cantilever. (b) The molecular structure of **DyP`**. The coordination environment around the Dy(III) remains the same as for **DyP**, however the side chains on the aryl groups are *tert*-butyl (as opposed to trihexylsilyl) and the pyrene anchor groups with dodecoxyl chains are tri-*iso*-propylsilyl groups, to promote crystal growth.

Supplementary Discussion

Additional Stability Diagrams

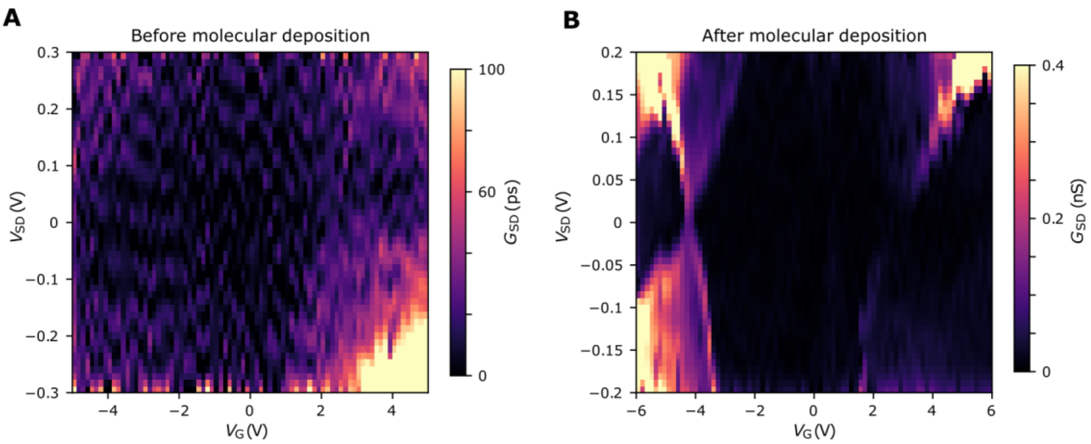

Supplementary Figure 24. Before and after **DyP** deposition conductance stability diagrams. Conductance stability diagrams over the accessible experimental gate range for the device presented in the main text before (**a**) and after (**b**) the **DyP** molecule was deposited onto the graphene nano-gap.

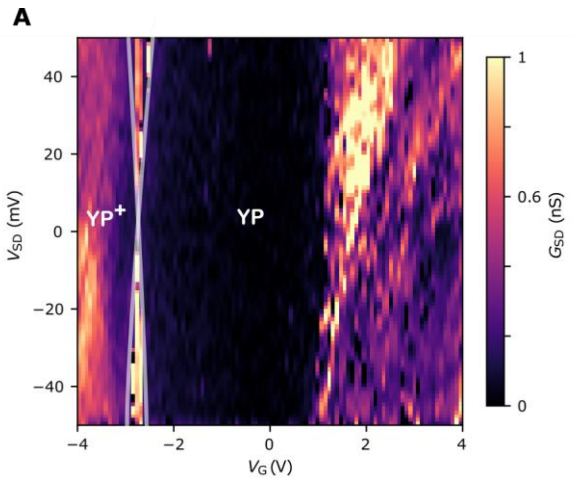

Supplementary Figure 25. Full **YP** conductance stability diagram. Conductance stability diagram over the entire measured gate range for the **YP** device presented in Fig. 3. The boundaries of the **YP**$^+$/**YP** transition at ~ -2.5 V_G are highlighted by white lines.

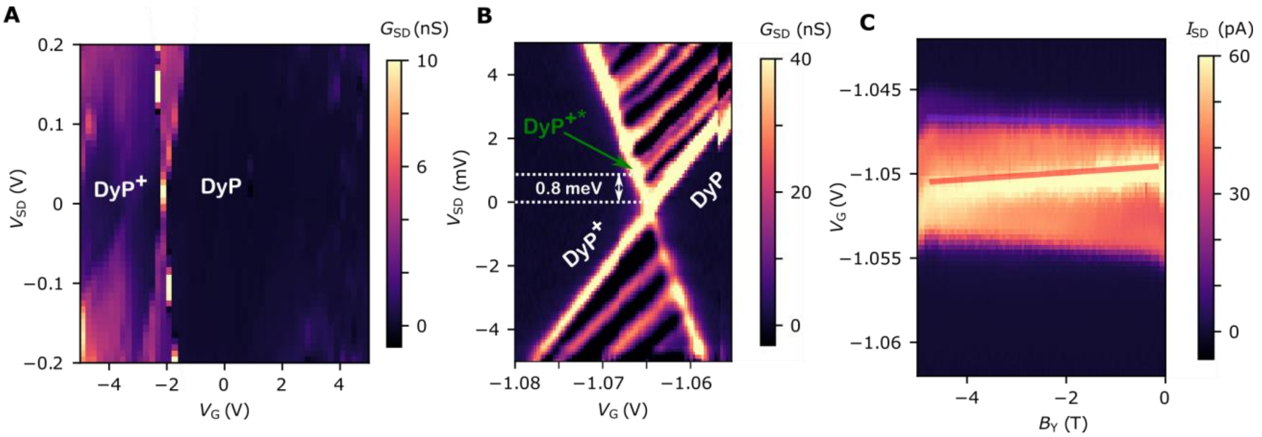

Supplementary Figure 26. Characterization of second **DyP** device. (**a**) Full conductance stability diagram of second **DyP** device across the entire accessible gate voltage range. A high resolution scan of the **DyP^{+}/DyP** transition is given in (**b**), a low lying excited state is again visible at 0.8 meV, along with other excited states, due to excitation of molecular vibrations or states within the graphene leads. Note that between the measurements of (**a**) and (**b**) the transition has shifted in V_G, likely due to a change in the occupancy of charge traps in the oxide substrate. (c) A measurement of the B_Y dependence of the $\mathbf{DyP}^{+}_{\uparrow\downarrow}$/ **DyP** ground-state to ground-state transition and the excited-state to ground state $\mathbf{DyP}^{+}_{\uparrow\uparrow}$/ **DyP** transition taken at V_{SD} = 1.5 meV. Data extracted from along the lines indicated on (**c**) are converted to energies and plotted in Fig. 4a.

<u>Determination of Molecule-Electrode Couplings</u>

For each device the coupling to the gate electrode, β, is determined from the slopes of the diamonds from the following equation:

$$^{1}/_{\beta} = {}^{1}/_{\alpha_+} - {}^{1}/_{\alpha_-} \qquad \text{(Supplementary Equation 1)}$$

where α_+ and α_- are the positive and negative slopes marking the edges of the Coulomb diamond respectively.[4] The total molecule-electrode coupling could then be extracted from the FWHM of a Lorentzian fit of the Coulomb peak divided by β.

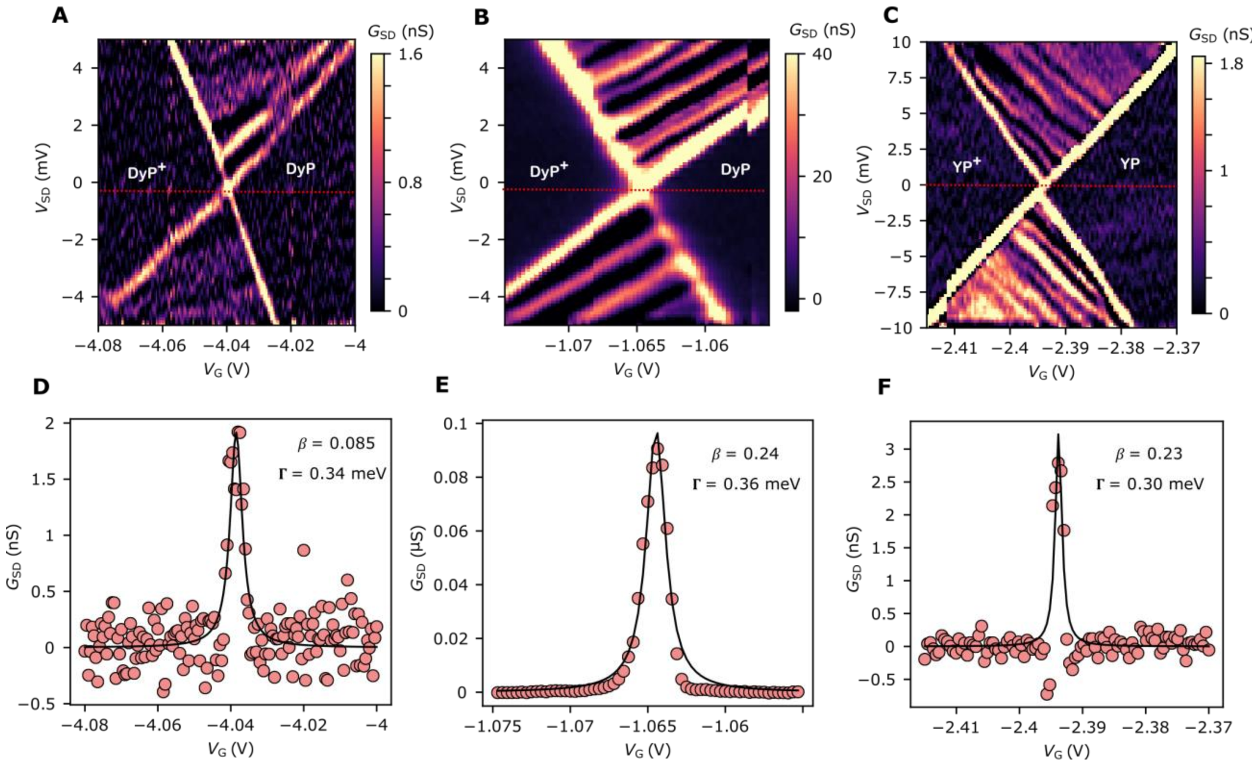

Supplementary Figure 27. Determination of molecule-electrode couplings. Conductance stability diagrams (**a**, **b**, **c**) of the devices **DyP** (main text), **DyP** (Supplementary) and **YP**, with the red line dotted line marking the zero-bias conductance measurements that are displayed in (**d**), (**e**) and (**f**). From the zero-bias traces we can extract the sum of the molecule-electrode couplings to the source and drain electrodes, $\Gamma = (\Gamma_S + \Gamma_D)$.

<u>Assignment of Molecular Oxidation States</u>

The molecular oxidation states in different Coulomb-blocked regions of a stability diagram can be assigned by measuring the magnitude of current in the sequential tunneling region that separates them.[5] The molecule-electrode coupling need to be sufficiently asymmetric, i.e., $\Gamma_S > \Gamma_D$ or $\Gamma_D > \Gamma_S$. In addition, the frontier orbitals involved in transport should be only spin-degenerate. As outlined in Ref [5], this leads to the four corners of the sequential tunneling region to have different magnitudes of current. The region where the magnitude of current is highest (given by the yellow triangle in Supplementary Fig. 28) is on the side (in terms of V_G) of the transition where the frontier orbital involved in transport is in the singly occupied state. For example, if the transition is **MP⁺/MP**, the highest current will be on the **MP⁺** side (at more negative V_G than the transition). If the transition is **MP/MP⁻** the highest current corner will be on the **MP⁻** side (at more positive V_G than the transition). We note that in the spin degeneracy in the HOMO of **DyP⁺** is lifted by exchange interactions with the Dy(III) ion, as is discussed in great detail in the main text. However as long as the current is measured at $|V_{SD}|$ much greater than the level splitting (0.8 meV), the assignment is valid.

The transitions from the 3 devices we study in this paper are assigned on this basis. For the **DyP** device presented in full in the main text, the highest-current corner is top left (Supplementary Fig. 28a), this indicates a **DyP⁺/DyP** transition and $\Gamma_D > \Gamma_S$. For the second **DyP** device, shown in Supplementary Fig. 26, the highest-current corner is again top left (Supplementary Fig. 28b), so it is a **DyP⁺/DyP** transition and $\Gamma_D > \Gamma_S$. For the **YP** device the highest current corner is bottom left (Supplementary Fig. 28c), so it is a **YP⁺/YP** and $\Gamma_S > \Gamma_D$.

For this analysis it was assumed that the closest transition to the Fermi level of the graphene (i.e. the closest transition to $V_G = 0$) was either **DyP⁺/DyP** or **DyP/DyP-**. It is possible that purely from the analysis above that the transitions in Supplementary Fig. 28 could be any odd/even transition such as

DyP^{3+}/DyP^{2+} or **DyP$^{–}$/DyP$^{2–}$**. However this would require the molecule to transfer at least two electrons to or from the substrate upon adsorption onto the graphene nanogap at V_G=0, which is unlikely. Furthermore, as the stability diagrams in Supplementary Fig. 24, 25 and 26 show, there is a large Coulomb-blocked region across the central portion of the full stability diagram for all the devices. This large Coulomb blocked region is due to a large addition energy for the *N* (MP) state. If we were studying higher oxidation states we would expect the neighboring transitions to be relatively close in potential on either side, however these are not observed experimentally. Therefore we are confident in the assignment of the molecular oxidation states as given on the stability diagrams.

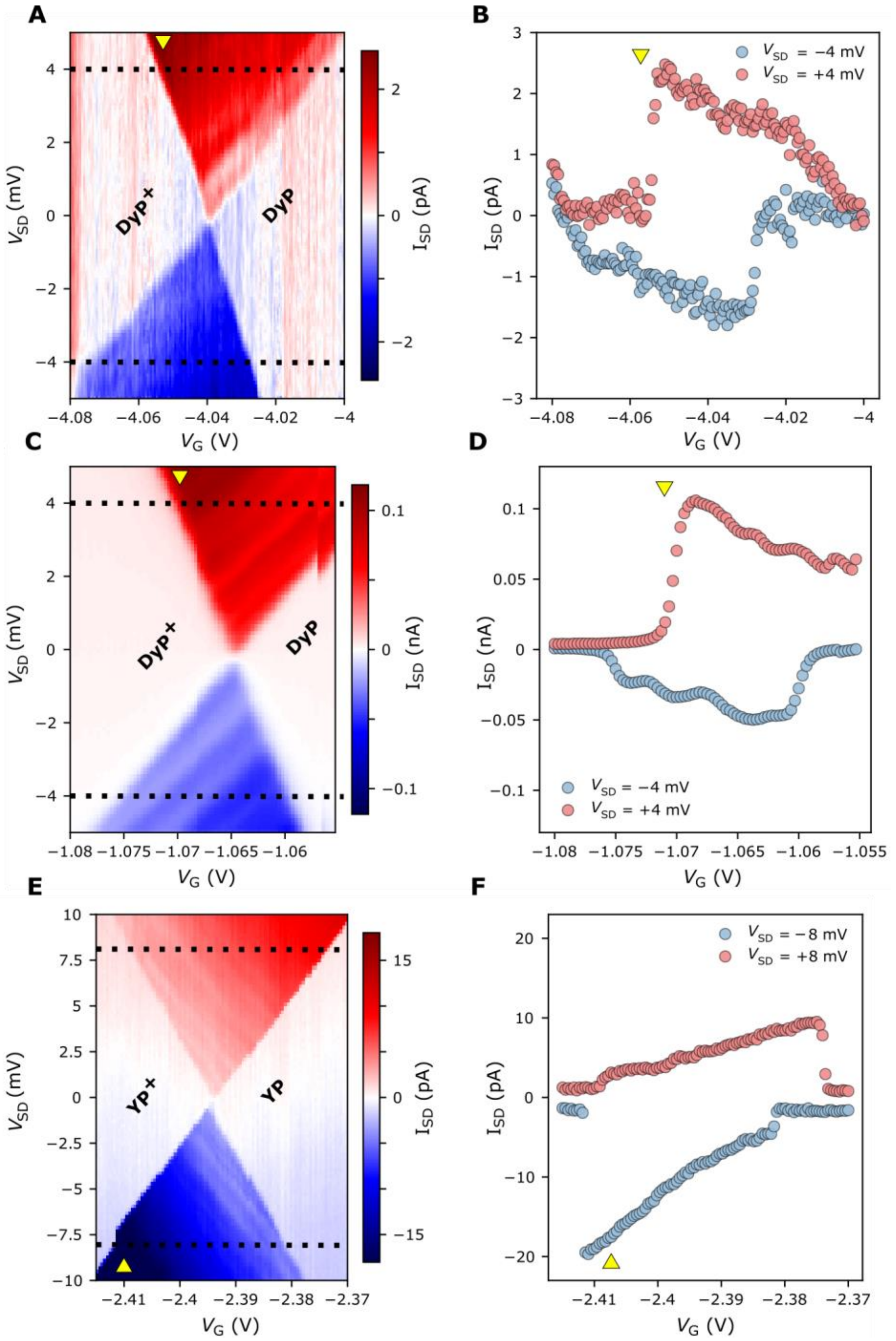

Supplementary Figure 28. Assignment of oxidation states. (**a**), (**c**), (**e**) display current stability diagrams of the 3 devices (**DyP**, **DyP**, and **YP**). The highest current corner of the sequential tunnelling region is indicated by the yellow triangle, and is top left, top left and bottom left for (**a**), (**c**), and (**e**), respectively. The gate traces displayed in (**b**), (**d**), and (**f**), display the magnitudes of the

current more clearly. The data gives the assignment of the molecular oxidation states in different Coulomb diamonds as marked on the stability diagrams.

Transport Simulation

In the following section the case of the $\mathbf{DyP^+}$/ $\mathbf{DyP}$ transitions that we observe experimentally are modelled within a rate-equation framework.[6] The three populations ($\boldsymbol{P}$) that need to be considered for the $\mathbf{DyP^+}$/ $\mathbf{DyP}$ transition are the neutral $\mathbf{DyP}$ and the ground and excited state of the cation, $\mathbf{DyP}^+_{\uparrow\downarrow}$ and $\mathbf{DyP}^+_{\uparrow\uparrow}$. The rate equations can be written compactly in matrix form, $d\boldsymbol{P}/dt = \boldsymbol{WP}$, explicitly:

$$\frac{d}{dt}\begin{pmatrix} P_{DyP_+(\uparrow\downarrow)} \\ P_{DyP_+(\uparrow\uparrow)} \\ P_{DyP} \end{pmatrix} = \begin{pmatrix} -\gamma^{DyP_+(\uparrow\downarrow)\to DyP} & 0 & \gamma^{DyP\to DyP_+(\uparrow\downarrow)} \\ 0 & -\gamma^{DyP_+(\uparrow\uparrow)\to DyP} & \gamma^{DyP\to DyP_+(\uparrow\uparrow)} \\ \gamma^{DyP_+(\uparrow\downarrow)\to DyP} & \gamma^{DyP_+(\uparrow\uparrow)\to DyP} & -(\gamma^{DyP\to DyP_+(\uparrow\uparrow)} + \gamma^{DyP\to DyP_+(\uparrow\uparrow)}) \end{pmatrix}\begin{pmatrix} P_{DyP_+(\uparrow\downarrow)} \\ P_{DyP_+(\uparrow\uparrow)} \\ P_{DyP} \end{pmatrix}$$

(Supplementary Equation 2)

Where $\gamma^{i\to j}$ is the rate of electron transfer from state i to j.

$$\gamma^{i\to j} = \sum_{l=S,D} \gamma_l^{i\to j} = \frac{\Gamma_l}{\hbar\pi}\int_{-\infty}^{\infty} f_l(\epsilon)k^{i\to j}(\epsilon)\,d\epsilon$$

(Supplementary Equation 3)

where Γ_l is the electronic coupling to the source and drain electrodes, $f_l(\epsilon)$ is the Fermi-Dirac distribution of electron energies in the leads, $k_l^{i\to j}(\epsilon)$ and is the energy-dependent molecular densities of states. In the absence of electron-vibrational coupling the form of the molecular densities of states is simply a Lorentzian centred on the potential of the $i \to j$ transition (ϵ_{ij}) and lifetime broadened by the molecule-electrode coupling:

$$k^{i\to j}(\epsilon) = \frac{\Gamma}{\Gamma^2 + (\epsilon - \epsilon_{i\to j})^2}$$

(Supplementary Equation 4)

The transition potentials, $\epsilon_{i\to j}$ depend on the source-drain and gate voltage, scaled by the coupling to the electrode:

$$\epsilon_{i\to j} = \epsilon^0_{i\to j} - \alpha_S V_{SD} - \beta V_G$$

(Supplementary Equation 5)

Under steady-state conditions that are experimentally relevant here, $d\boldsymbol{P}/dt = \boldsymbol{WP} = 0$, and the populations are of $P_{DyP_+(\uparrow\downarrow)}$, $P_{DyP_+(\uparrow\uparrow)}$ and P_{DyP} are obtained as the null space of $\boldsymbol{W}$. The net current through the molecule junction can be obtained by considering electron-transfer at one electrode:

$$I_{SD} = e[(\gamma^{DyP\to DyP_+(\uparrow\downarrow)} + \gamma^{DyP\to DyP_+(\uparrow\uparrow)})P_{DyP} - (\gamma^{DyP_+(\uparrow\downarrow)\to DyP}P_{DyP_+(\uparrow\downarrow)} + \gamma^{DyP_+(\uparrow\uparrow)\to DyP}P_{DyP_+(\uparrow\uparrow)})]$$

(Supplementary Equation 6)

where e is the electron charge.

The combination of these formulae allow the calculation of the stability diagram, as shown in Supplementary Figure 29.

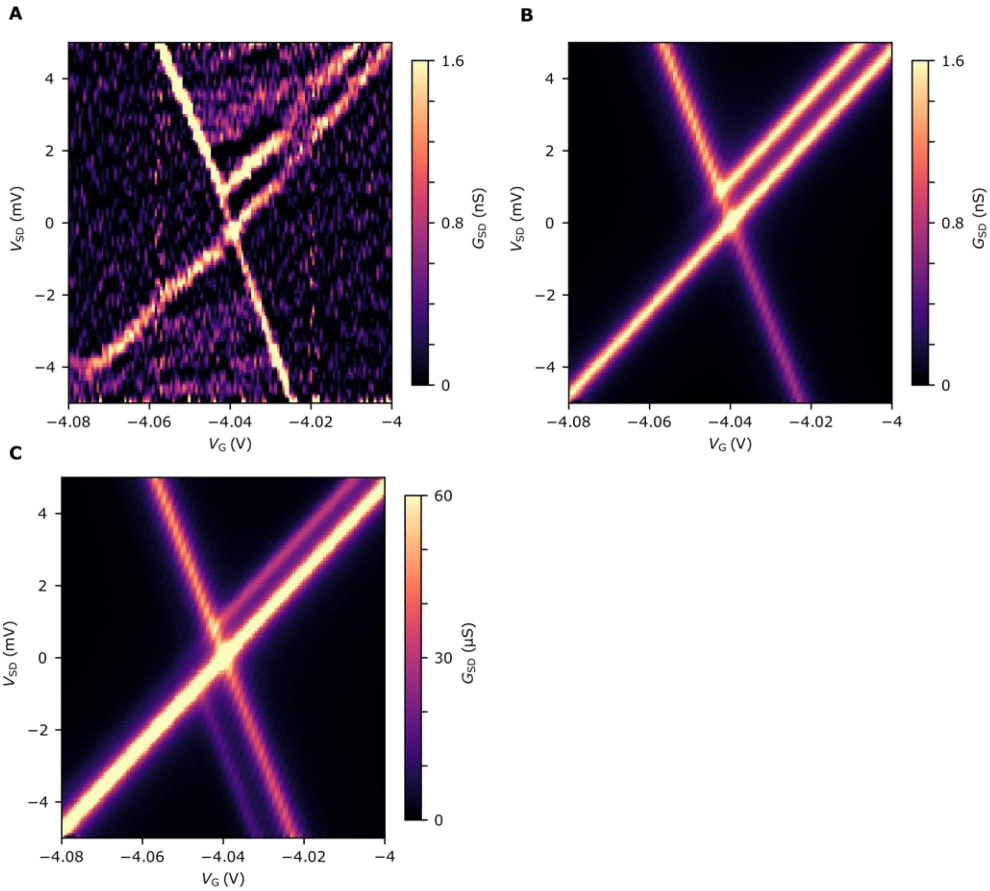

Supplementary Figure 29. Simulation of experimental data. Asymmetric molecule-electrode coupling causes the excited state transition to only appear at positive V_{SD}. (**a**) Experimental conductance stability diagram of **DyP**. Simulation of the experimental data with $\Gamma_D \gg \Gamma_S$ (**b**) and $\Gamma_D = \Gamma_S$ (**c**).

Spin Polarization

On the basis of the model presented above, we can separate the tunneling current through the device at positive V_{SD} into the contributions that occur *via* the AFM (↑↓) and FM (↑↑) states of **DyP**$^{+}$. At positive bias and with the asymmetric molecule electrode coupling observed, $\Gamma_D \gg \Gamma_S$, Supplementary Equation 7 reduces to:

$$I_{SD} = e\left(\gamma^{DyP\to DyP_{+}(\uparrow\downarrow)} + \gamma^{DyP\to DyP_{+}(\uparrow\uparrow)}\right) \quad \text{(Supplementary Equation 7)}$$

$$I_{SD} = I_{\uparrow\downarrow} + I_{\uparrow\uparrow} \quad \text{(Supplementary Equation 8)}$$

Therefore the total current is the sum of the current, $I_{\uparrow\downarrow}$, that flows *via* the $\mathbf{DyP}^{+}_{\uparrow\downarrow}$ state and $I_{\uparrow\uparrow}$ the current that flows *via* $\mathbf{DyP}^{+}_{\uparrow\uparrow}$. Due to the Pauli Exclusion Principle $I_{\uparrow\downarrow}$ and $I_{\uparrow\uparrow}$ are pure spin-polarised currents, as shown in Supplementary Figure 30b. At B > 0 as AFM ground state is: $|-½, +½\rangle$. $I_{\uparrow\downarrow}$ is when the m_s = +1/2 electron is the only mobile electron on the molecule, and conduction occurs through the device by tunneling on and off of a polarized current of m_s = +1/2 electrons. Conversely when B < 0 the opposite is the case and we can generate a spin-polarized current of m_s = –1/2 electrons. The spin-polarization, P, is the fraction of the total tunneling current that is spin-polarised:

$$P = \frac{I_{\uparrow\downarrow} - I_{\uparrow\uparrow}}{I_{\uparrow\downarrow} + I_{\uparrow\uparrow}} \quad \text{(Supplementary Equation 9)}$$

By inspection of Supplementary Equations 7 and 8 it is shown that, I_{SD}-V_G traces are the sum of the integrals over two lifetime-broadened Lorentzian functions $k^{i\to j}$, one for $\mathbf{DyP}^{+}_{\uparrow\downarrow}$/ **DyP** entering the bias window to give $I_{\uparrow\downarrow}$, and a second for $\mathbf{DyP}^{+}_{\uparrow\uparrow}$/ **DyP** entering the bias window to give $I_{\uparrow\uparrow}$. dI_{SD}/dV_G *vs* V_G can be fitted with two Lorentzians that are split by the intramolecular exchange splits so they can be fitted and integrated separated. Integration over these two Lorentzian gives $I_{\uparrow\downarrow}$ and $I_{\uparrow\uparrow}$. The ratio P vs V_G is calculated from these and displayed in Supplementary Fig. 30c, but only when the signal-to-noise ratio (I_{SD}/I_{noise}) is greater than 1. I_{noise} is calculated as the root-mean-squared (RMS) value of the current in a Coulomb-blocked region of the same dataset.

At B = 0.08 T we calculate P as 0.94 ± 0.35 at V_G = -4.029 V. The range over which the spin polarisation ratio is over 0.90 with an error below 0.40 is ~ 4 meV. As B_Y increases to 5 T the spin polarisation of the current is similar within the error, P as 0.97 ± 0.28 at V_G = -4.027 V, however the range over which the value is over 0.90 with an error of less than 0.40 is 8 meV, due to Zeeman splitting further separating the energies of the transitions.

The error, ΔP, on P is calculated as follows, where $\Delta I_{\uparrow\downarrow} = \Delta I_{\uparrow\uparrow} = I_{noise}$:

$$\Delta P^2 = \left(\Delta I_{\uparrow\downarrow}\left(\frac{\partial P}{\partial I_{\uparrow\downarrow}}\right) + \Delta I_{\uparrow\uparrow}\left(\frac{\partial P}{\partial I_{\uparrow\uparrow}}\right)\right)^2 \quad \text{(Supplementary Equation 10)}$$

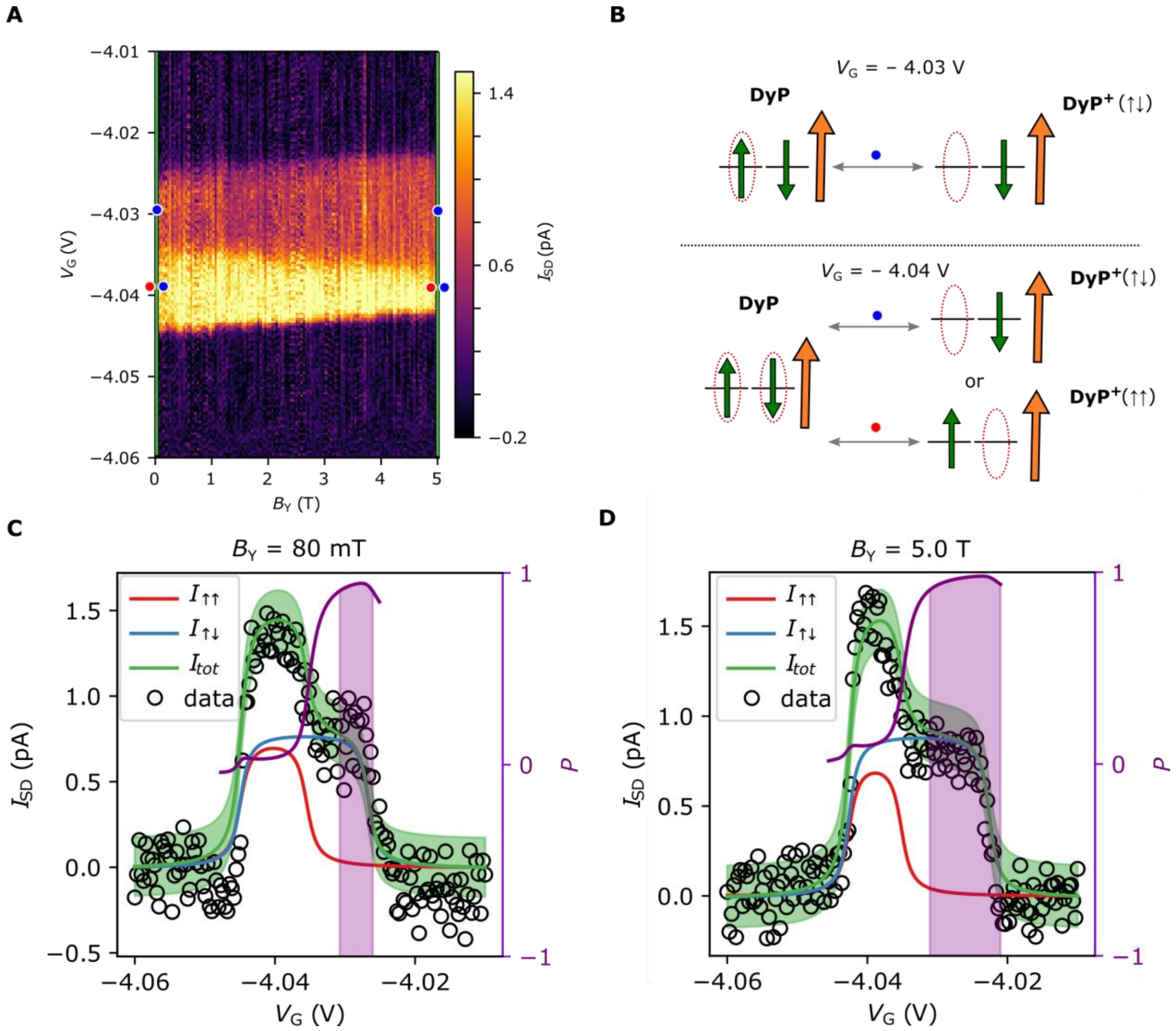

Supplementary Figure 30. Calculation of spin polarization in DyP. The dataset of I_{SD}-V_G-B_Y at V_{SD} = 1.5 mV is displayed in (**a**). The green lines indicate the data displayed as open circles in (**c**) and (**d**) at B_Y = 80 mT and B_Y = 5 T respectively. Transport via **DyP**$^{+}_{\uparrow\downarrow}$/ **DyP** and **DyP**$^{+}_{\uparrow\uparrow}$/ **DyP** channels are modelled in red and blue respectively, (each one is the integral over a Lorentzian for when the state enters the bias window). The sum is given in green, the shaded green region is the error on the current. The ratio of P is plotted in (**c**) and (**d**) in the regions where $SNR > 1$ (I_{noise} = 0.12 pA), otherwise it is set to zero. The shaded purple area is the V_G range over which the ratio $P > 0.9$ and $\Delta P < 0.3$, and widens with increasing B_Y.

Supplementary Tables

**Energy (cm^{-1})**	$\boldsymbol{g_x}$	$\boldsymbol{g_y}$	$\boldsymbol{g_z}$	**Angle relative to ground $\boldsymbol{g_z}$ (°)**
0.00	0.10	0.16	17.62	--
20.90	0.20	0.58	15.58	41.86
91.83	1.70	2.75	10.92	30.92
121.85	0.32	1.54	17.02	18.98
191.03	1.27	2.88	10.17	32.98
244.95	2.90	6.68	11.60	80.83
477.33	0.41	7.94	10.88	87.26
496.15	0.35	8.51	11.34	76.57

Supplementary Table 1. Eigenstates of ground $^6H_{15/2}$ multiplet for **DyP** from ab initio CASSCF-SO calculations.

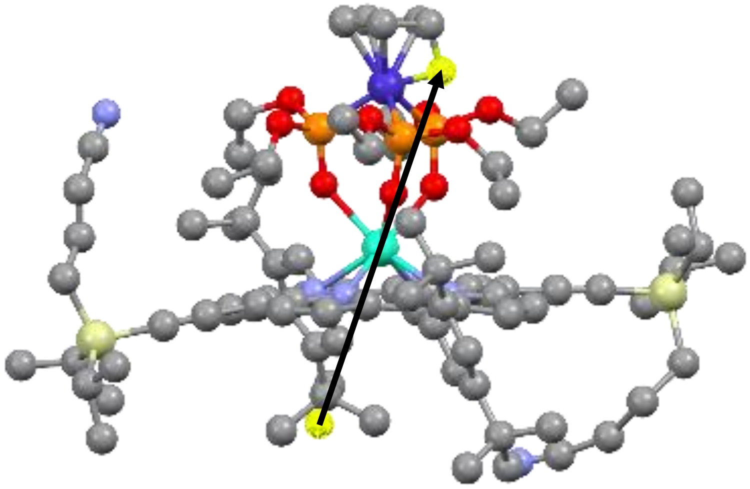

Supplementary Figure 31. CASSCF-SO-calculated magnetic easy axis (black arrow between yellow balls) for ground Kramers doublet in **DyP**.

	A	C_0	C_1	C_2	C_3	C_4
**DyP$^+$**	0.444	–0.128	0.151	–0.020	–0.069	–0.003
**DyP^{+*}**	0.875	0.936	0.034	–0.038	–0.028	0.002

Supplementary Table 2. Parameters from fitting experimental data to: $E = A\sum_{n=0}^{N} C_n \cos(2n\vartheta)$. The inclusion of additional fitting parameters (i.e. C_5 onwards) did not decrease the fitting errors.

Supplementary References

1. Limburg, B., *et al.* Anchor groups for graphene-porphyrin single-molecule transistors. *Adv. Funct. Mater.* **28,** 1803629 (2018).

2. Kläui, W. The Coordination Chemistry and Organometallic Chemistry of Tridentate Oxygen Ligands with π-Donor Properties. *Angew. Chem., Int. Ed. Engl.* **29,** 627-637 (1990).

3. Grozema, F. C., *et al.* Supramolecular Control of Charge Transport in Molecular Wires. *J. Am. Chem. Soc.* **129,** 13370-13371 (2007).

4. Osorio, E. A., *et al.* Single-molecule transport in three-terminal devices. *Journal of Physics: Condensed Matter* **20,** 374121 (2008).

5. Limburg, B., *et al.* Charge-state assignment of nanoscale single-electron transistors from their current–voltage characteristics. *Nanoscale* **11,** 14820-14827 (2019).

6. Sowa, J. K., Mol, J. A., Briggs, G. A. D., Gauger, E. M. Beyond Marcus theory and the Landauer-Büttiker approach in molecular junctions: A unified framework. *J. Chem. Phys.* **149,** 154112 (2018).
